# An ethnobotanical study of medicinal plants in Mana Angetu District, southeastern Ethiopia

**DOI:** 10.1186/1746-4269-4-10

**Published:** 2008-04-28

**Authors:** Ermias Lulekal, Ensermu Kelbessa, Tamrat Bekele, Haile Yineger

**Affiliations:** 1 Department of Biology, Debre Berhan University, P.O. Box 445, Debre Berhan, Ethiopia; 2 National Herbarium, Addis Ababa University, P. O. Box 3434, Addis Ababa, Ethiopia; 3 Department of Biology, Jimma University, P. O. Box 5195, Jimma, Ethiopia

## Abstract

This study documents indigenous medicinal plant utilization, management and the threats affecting them. The study was carried out in Mana Angetu district between January 2003 and December 2004. Ethnobotanical data were collected using semi structured interviews, field observations, preference and direct matrix ranking with traditional medicine practitioners. The ethnomedicinal use of 230 plant species was documented in the study area. Most of the plants (78.7%) were reportedly used to treat human diseases. The most frequently used plant part were roots (33.9%), followed by leaves (25.6%). Most of the medicinal species (90.4%) were collected from the wild. Direct matrix analysis showed that *Olea europaea *L. Subsp. *cuspidata *(Wall. ex G. Don) was the most important species followed by *Acacia tortilis *(Forssk.) Hayne (120) indicating high utility value of these species for the local community. The principal threatening factors reported were deforestation (90%), agricultural expansion (85%) and fire (53%). Documenting the eroding plants and associated indigenous knowledge can be used as a basis for developing management plans for conservation and sustainable use of medicinal plants in the area.

## Background

Since ancient times plants have been indispensable sources of both preventive and curative traditional medicine preparations for human beings and livestock. Historical accounts of traditionally used medicinal plants depict that different medicinal plants were in use as early as 5000 to 4000 BC in China, and 1600 BC by Syrians, Babylonians, Hebrews and Egyptians [1]. Much of an indigenous knowledge system, from the earliest times, is also found linked with the use of traditional medicine in different countries [2]. Traditional medicine refers to any ancient, culturally based healthcare practice different from scientific medicine and it is commonly regarded as indigenous, unorthodox, alternative or folk and largely orally transmitted practice used by communities with different cultures [3]. WHO also defined traditional medicine as health practices, approaches, knowledge and beliefs incorporating plant, animal and mineral based medicines, spiritual therapies, manual techniques and exercises applied to treat, diagnose and prevent illnesses or maintain well being [4].

Beside their use in fighting various ailments at local level different medicinal plants are used as export commodities, which generate considerable income [5]. These plants are normally traded in dried or freshly preserved form as whole or comminuted [6]; and their global markets are found in China, India, Germany, France, Italy, Japan, England and USA [7]. Currently, large number of medicinal plants has found their way as raw materials of modern bio-pharmaceutical industry.

In Ethiopia the long history of using traditional medicinal plants for combating various ailments can be confirmed by referring to the medico-religious manuscripts in the country [8]. Plant remedies are still the most important and sometimes the only source of therapeutics for nearly 80% of the population in Ethiopia [9]. The current loss of medicinal plants in the country due to natural and anthropogenic factors links with the missing of valuable indigenous knowledge associated with the plants. This strong link suggests a need to conduct ethnobotanical research and to document the medicinal plants and the associated indigenous knowledge. Such studies are useful to identify threatened plants and to take appropriate conservation measures. The present research documents the wealth of indigenous knowledge on utilization, management and conservation of medicinal plants as well as the threats to the plants in Mana Angetu District, Southeastern Ethiopia.

## Materials and methods

### Study area and ethnographic background of the local people

**Figure 1:**
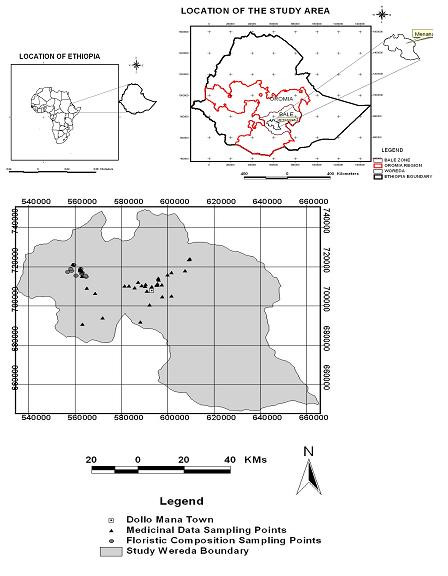
Map of the study area showing medicinal data sampling points.

Mana Angetu District is found in the Southwestern corner of Bale Zone, Oromia National Regional State, Ethiopia (Figure 1). The district is located between 06°10' N and 06°31' N, and 039°30' E and 039°45' E. The highland portion of the district is an extension of the Bale massif. Geologically the Bale Mountains appear to be of volcanic origin resulting from the trappean lava, which cover the Mesozoic strata [10].

Based on the meteorological data recorded at Dollo Mana station for 18 years (January1986 to December 2003), the study area had bimodal rainfall distribution with the highest rain falling from March to May and then in October. The mean annual rainfall of the study area was 740.47 mm, and the mean annual temperature was 30.57°C.

The vegetation type of the area is moist montane forest type, which is also found in Southwestern part of Ethiopia [11]. It is composed of various species of plants including the largest and commercially most important trees in Ethiopia like *Pouteria adolfi-friederici *(Engl.) Baehni, *Podocarpus falcatus *(Thunb.) Mirb. and *Polyscias fulva *(Hiern) Harms; the under storey bears coffee plants (*Coffea arabica *L.).

A total population of approximately 120,000 inhabits the district. About 91% of the inhabitants are engaged in agriculture; of which 28% conduct crop production, 18% cattle rearing and 45% mixed farming. The district has only two health centers and three clinics supporting the entire population. The livestock population of 220,000 are supported by three veterinary clinics and six health workers [12].

The majority of the local people in Mana Angetu District of Bale Zone belong to the Oromo ethnic group. The Oromo people constitute at least 40% of the Ethiopian population [13] and are traditionally pastoral tribes. They are linguistically Cushitic speaking, using the widely spoken *Afaan Oromo *language. Some Oromo people practice sedentary agriculture while many others practice mixed farming, which involves cultivation of crops and livestock rearing. According to [14], they probably originated from North Somalia and later migrated to Lake Turkana and the Ethiopian highlands. In contrast other publications [15] state that the Oromo ethnic groups are from the highlands of Bale, Borana and Guji in Southeastern Ethiopia. The people had their own religion called *Waaqeffannaa *before the expansion of Christianity and Islam [16]. This religion is still maintained by a few of the Oromo people [17]. They have a long tradition of age based social organization called the *Gada *system by which they maintain their social, political and belief systems.

### Methods

Eighteen Kebeles, which are found as buffer zones at the southern part of the Bale Mountains National Park, were selected for ethnobotanical data collection. A total of 70 informants (63 males and 7 females) were selected purposively following [18] with the help of local administrators, the office of the district's traditional healers association and local elderly people. Nominations on knowledge depth of respondents were collected from local elderly people, heads of the district's traditional healers association and the local administrators of each Kebele. Similar responses obtained from the three groups were used to identify knowledgeable traditional healers. The traditional healers identified were asked for their consent to share their knowledge only for the purpose of this study.

The methods used for ethnobotanical data collection were semi structured interviews described by [3] and [18]; field observation, preference ranking and direct-matrix ranking according to [3,18,19]. The respondents' background, health problems treated, diagnosis and treatment methods, local name of medicinal plants used, source of collection (wild/cultivated), growth form, degree of scarcity, plant part used, methods of preparation and application, threats to medicinal plants and conservation practices of respondents were carefully recorded. Observations were made on the morphological features and habitats of each medicinal plant species in the field.

The frequency of citation for each of the reported ailments was used as a basis to identify the most frequently occurring human ailment in the study area, which in this case was gonorrhoea. Preference ranking of all the medicinal plant species (7 species) reported by traditional healers to treat this ailment was conducted following [18] and [19] so as to show healers' perception on the relative degree of efficacy of the 7 species and get useful information to identify target plants for future pharmacological investigations. The key informants for purposes of ranking these species were selected randomly from among all informants who reported to treat the ailment.

The status of all the medicinal plants was recorded as abundant, less abundant, rare or very rare as per healers' perception during the semi structured interviews. To determine the most threatened medicinal plants in the study area, authors short listed all medicinal plant species (6 species) reported by traditional healers as very rare in the area and conducted preference ranking of these species using 14 randomly selected informants. Direct matrix ranking exercise was conducted for seven multipurpose medicinal plants to determine the main cause for over harvesting of the respective plants. Frequency of citation as multipurpose species was used as a criterion to select the seven candidate medicinal plant species for the direct matrix ranking exercise. Additional file 1 shows pictures reflecting some of the field data collection events in this study.

Identification of the medicinal plant specimens collected from the study area was performed at the National Herbarium (Ethiopia), Addis Ababa University using taxonomic keys and Floras [20-26] and by comparison with already identified herbarium specimens. The identified specimens were deposited at the National Herbarium.

### Data analyses

Ethnobotanical data were entered in to Excel spreadsheet and summarized using descriptive statistics [27]. The spreadsheet data filter facility was employed to determine frequencies of citations so as to identify the most common ailments in the study area, popularly used medicinal plant species and multipurpose plant species, to determine proportions of different variables like plant families, growth forms, source of collection, degree of scarcity, plant part used, methods of preparation and threatening factors. The preference values/scores assigned by key informants for selected medicinal plant species were added and ranked during the preference ranking and direct matrix ranking activities.

## Results and discussion

### Medicinal plants in Mana Angetu District

In this study a total of 230 medicinal plant species used for treatment of human and livestock ailments were collected (see Additional file 2). Of these, 181 (78.70 %) were used as human medicine, 27 (11.74%) as livestock medicine and the remaining 22 (9.57 %) were used for treating both human and livestock ailments. The presence of such a large number of medicinal plant species and associated ethnomedicinal knowledge in the district compared to number of species reported for other regions in Ethiopia [see [28-34]] indicates that the area has a very high diversity of medicinal plant species and is a site for various indigenous knowledge.

The medicinal plants collected belong to 177 genera and 74 families. The family Fabaceae was represented by the highest number of species (26 species, 11.3%). This was followed by Asteraceae (19 species, 8.3%), Euphorbiaceae (15 species, 6.5%), Asclepiadaceae (11 species, 4.8%), and Rubiaceae (9 species, 3.9%). Families Lamiaceae, Acanthaceae, Rutaceae, and Verbenaceae were represented by 7 species each, where as Malvaceae, Solanaceae and Vitaceae with 6 species each. This also indicates that the area consisted of considerable diversity of plant species. These plant families are consistently recorded in different ethnomedicinal inventories [for example, [31-36]], which could be attributed to their wider distribution and abundance [35] and rich bioactive ingredient contents [37].

**Figure 2:**
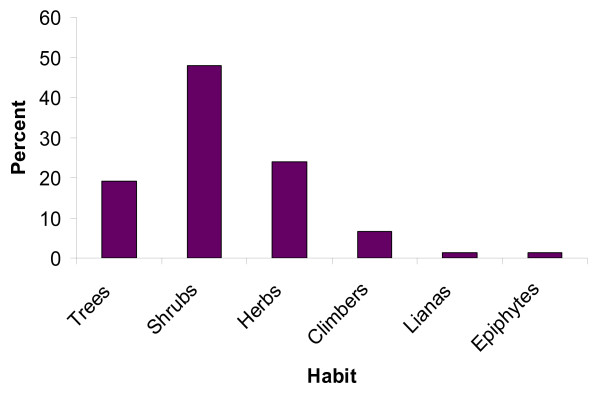
Growth forms (habits) of the reported ethnomedicinal plant species.

The results of growth form analysis of medicinal plants showed that shrubs made up the highest proportion being represented with 110 species (47.83 %), followed by herbs (55 species, 23.91 %), trees (44 species, 19.13 %), climbers (15 species, 6.52 %), lianas, and epiphytes (3 species each, 1.30 % each) (Figure 2). This finding is contrary to the general pattern seen in most medicinal inventories [for example, [28,31,32]] where herbaceous medicinal plants dominate. This could be associated to the abundance and year round availability of shrub species in the study area.

Of the 230 medicinal plants studied, 208 species (90.43 %) were collected from the wild while 13 species (5.65 %) were found in cultivation and 9 species (3.91 %) were obtained both from cultivation and the wild. This indicates that the practitioners depend on the wild source or the natural environment rather than home gardens to obtain the medicinal plants, and the activity of cultivating medicinal plants is very poor in the study area. It also indicates that the natural forest of Mana Angetu is being over exploited by traditional practitioners for its medicinal plants composition.

The plant parts used widely to treat human and livestock health problems include root, stem, leaves and others (Table 1). The most commonly used plant parts for herbal preparations in the area were roots (33.91 %) and leaves (25.65 %). Such wide harvesting of roots, which are important for survival of plants has a negative influence on the survival and continuity of useful medicinal plants and hence affects sustainable utilization of the plants. Large proportion of herbal prescription from root sources was also reported by [32] and [38] in their ethnobotanical investigations.

**Table 1:** Medicinal plant parts used by traditional healers for remedy preparation

**Plant part used**	**No of species**	**Percent**
Root wood	78	33.9
Leaves	59	25.7
Root and leaves	30	13
Root bark	15	6.5
Seeds	7	3
Stem bark	6	2.6
Stem bark, Root bark	5	2.2
Fruits	4	1.7
Stem wood	3	1.3
Root, stem and leaves	3	1.3
Stem and leaves	3	1.3
Root and stem	3	1.3
Leaf, fruit and root	2	0.9
Latex only	2	0.9
Leaves, seed and stem	2	0.9
Root and fruit	2	0.9
Leaves and stem bark	2	0.9
Resin and root	2	0.9
Fruit and leaves	1	0.4
Root, leaves, seeds, fruit and oil	1	0.4

Total	230	100.00

The medicinal plants have various methods of preparation and application for different types of ailments and they have various preparation forms like concoction, decoction, powder, and crushed and homogenized in water. Concoction (60 species, 26.1%) constituted the highest type of preparation form, followed by crushed and homogenized in water (46 species, 20 %) and powder form (37 species, 16.1%). The preparation and application methods vary based on the type of disease treated and the actual site of the ailment.

**Figure 3:**
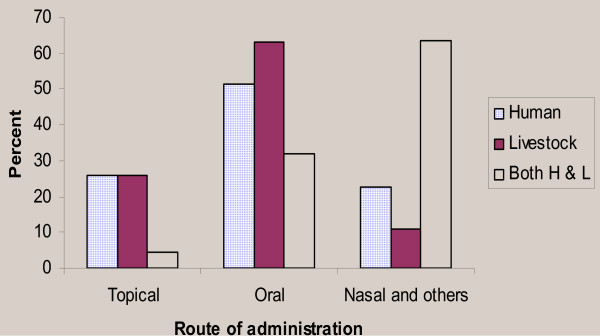
Reported routes of administration of medicinal plant remedies used for human beings, livestock and both.

The medicinal plant preparations were applied through different routes of administration like oral, topical or dermal, and nasal routes. However, oral application (116 preparations, 50.43 %) was the highest and most commonly used route of application followed by topical or dermal application (54 preparations, 23.48 %) (Figure 3). These results are consistent with the findings of various ethnobotanical researches elsewhere in Ethiopia, such as that of [[28,30,32], and [33]].

Most of the medicinal plant species collected and identified in this study were also medicinally used in other parts of Ethiopia and also other African countries. For example, of the 230 medicinal plants found and used in Mana Angetu District: [39] documented 24 species, [40] documented 59 species; [41] documented 41 species; [38] documented 19 species; and [29] documented 25 species, as medicinally important to cure human and livestock diseases. In Uganda 21 species were found documented in the medicinal plant list of [42], and in Kenya 20 species were documented in the list of [43]. Such widespread use of these plants by different groups of societies in different countries could to a certain extent be attributed to their efficacy. In other words, the ethnomedicinal reports of those species from wider geographical regions and different cultural groups could validate the medicinal properties of the species.

### Disease types, treatment methods and herbal preparations used to treat human health problems

Though more than 50 different disease types were recorded as human health problems in the district, the major and most widespread diseases according to the informants include gonorrhoea, jaundice, kidney infection and general malaise (Table 2). In addition to these the practitioners were also visited for diseases like skin infections, mental disorder, rheumatoid arthritis and hemorrhoids.

**Table 2:** Common diseases affecting human health in Mana Angetu District

**Disease type (local name)**	**Frequency**
Gonorrhoea (*Chobto*)	22
Jaundice (*Alati*)	11
Kidney (*Birbirti*)	11
General malaise (*Michi*)	9
Skin infection (*Sibiji*)	8
Mental disorder (*Merata*)	7
Rheumatoid arthritis (*Qilensa*)	5
Hemorrhoid (*Qormade*)	5

The result as shown in Table 2 indicates that gonorrhoea was the most common disease followed by jaundice and kidney infections for which patients commonly visit the traditional medical practitioners (TMPs).

Interviews with the TMPs found different diagnosis and treatment methods depending on the type of the ailment. The practitioners commonly diagnose each health problem by an interview and visual inspection of the patient. Patients or their attendants are commonly interviewed for symptoms observed and the duration of the health problem. Changes in eye and skin color, tongue and throat regions, body temperature and status of sores are all visually inspected by the practitioner and the remedy is prescribed.

Internal ailments were commonly treated by making the patient drink herbal preparations; skin infections such as ringworm were treated by rubbing and painting herbal preparations on an infected skin; sores by chewing and spitting remedial plant part on the sore; headaches and fever by steam bath and vapor inhalation. Similar results were reported elsewhere in Ethiopia by [29] and [41].

Though special care was taken, some herbal preparations had side effects and resulted in diarrhoea and vomiting. When such conditions happened, antidotes like coffee, milk, honey, yogurt, butter and powder of roasted barley were used or ordered by most of the practitioners to reverse the condition. Most of the medicinal plant preparations given did not have standardized doses. In most cases dosages were determined according to the age, sex and physical appearance of the patient. Some of the medicinal plant preparations were measured in a small cup, a jug, while others as handful, or spoonful. Proper care is needed for sanitation of herbal preparations and their containers. Some preparations were placed in unclean containers and areas which may result contamination and seriously affect users when drunk. Patients suffered from overdose and contaminations were believed to recover by application of antidotes.

Among the medicinal plants used for herbal preparations to treat human health problems, *Cissampelos pariera *L. was found commonly used by most of the traditional practitioners followed by *Carissa spinarum *L.*, Withania somnifera *(L.) Dun., *Croton macrostachyus *Del. and *Euclea divinorum *Hiern (Table 3).

**Table 3:** Popularly used medicinal plants of the Mana Angetu District

**Scientific name**	**Frequency of report**
*Cissampelos pariera *L	51
*Carissa spinarum *L.	33
*Croton macrostachyus *Del.	14
*Euclea divinorum *Hiern	14
*Withania somnifera *(L) Dun.	14
*Rubus steudneri *Schwinef.	10
*Psychotria orophila *Petit	9
*Senna occidentalis *(L.) Link	9
*Warburgia ugandensis *Sprague	8
*Ximenia americana *L.	8

### Preference ranking of medicinal plants used for treating gonorrhoea

Preference ranking of 7 medicinal plants that were reported as effective for treating gonorrhoea, was conducted after selecting 10 key informants. The informants were asked to compare the given medicinal plants based on their efficacy, and to give the highest number (7) for the medicinal plant which they thought most effective in treating gonorrhoea and the lowest number (1) for the least effective plant in treating gonorrhoea. As shown in Table 4, *Euclea divinorum *scored the highest mark and ranked first indicating that it was the most effective in treating gonorrhoea followed by *Ricinus communius *L. Ethnobotanical investigations done elsewhere in Ethiopia also reported that *Euclea divinorum *was used for treatment of gonorrhoea [29,40]. The compounds lupeol, lupene, betulin, 7-methyljuglone, isodiospyrin, shinalone, catechin and 3fl-(5-hydroxyferuloyl)lup-20(30)-ene were isolated by [44] from the root bark of this plant species. Antiperiodontopathic bacterial activity of this medicinal plant species was reported by [45]. Further pharmacological test of this species against gonorrhoea might reveal promising results.

**Table 4:** Preference ranking of medicinal plants used for treating gonorrhoea

	**Informants labeled A to J**											
	
**Medicinal plants**	**A**	**B**	**C**	**D**	**E**	**F**	**G**	**H**	**I**	**J**	**Total score**	**Rank**
*Acokanthera schimperi *(A. DC.) Schweinf.	4	7	5	4	7	5	2	1	2	1	38	**4**
*Crabbea velutina *S. Moore	2	5	4	2	4	7	3	2	3	2	34	**5**
*Euclea divinorum *Hiern	7	6	6	7	5	3	5	6	4	7	55	**1**
*Gnidia stenophylla *Gilg	3	3	3	6	6	4	6	3	6	4	44	**3**
*Ricinus communis *L.	5	4	7	5	1	6	4	5	5	5	47	**2**
*Solanum incanum *L.	1	2	2	3	2	1	1	7	7	6	32	**6**
*Surregada procera *(Prain) Croizat	6	1	1	1	3	2	7	4	1	3	29	**7**

**Key**-Scores in the table indicate ranks given to medicinal plants based on their efficacy. Highest number (7) for the medicinal plant which informants thought most effective in treating gonorrhoea and the lowest number (1) for the least effective plant.

### Medicinal plants used to treat livestock health problems

The TMPs use different forms of remedy preparations and applications to treat livestock diseases. The most popular and widely used medicinal plants used to treat livestock diseases in the study area include *Acacia seyal *Del., *Breonadia salicina *Vahl (Hepper and Wood), and *Rhyncosia ferruginea *A. Rich. Black leg/*aba gorba*, scabies/skin infection and diarrhoea/*tuma *were also reported as the most common livestock health problems. Most of the preparations used (12 species, 44.44%), involved crushing and homogenizing the remedies in water, followed by concoction (9 species, 33.33%) and powdering (4 species, 14.81%).

Based on the nature of the ailment the remedies were applied through different routes. Oral application of remedies was found the highest (62.96%), followed by topical application (25.93%) and nasal application (11.11%).

### Ranking of threatened medicinal plants

Based on the degree of threat and rarity, ranking of 6 different medicinal plants that were recorded for rarity, was conducted after selecting 14 key informants in the study area. The results showed that *Withania somnifera *got the highest score indicating that it is the most threatened plant followed by *Asparagus africanus *Lam. and *Dioscorea quartiniana *A. Rich. On the other hand *Pittosporum viridiflorum *Sims scored the least indicating that it was less threatened when compared to the other species (Table 5).

**Table 5:** Ranking of medicinal plants reported as threatened in the study site

**List of medicinal plants**	Key informants coded A-N															
	
	**A**	**B**	**C**	**D**	**E**	**F**	**G**	**H**	**I**	**J**	**K**	**L**	**M**	**N**	**Total score**	**Rank**
*Olea europaea L. subsp. cuspidata (Wall. ex G.Don)*	3	2	1	4	1	1	2	3	6	5	5	2	4	2	41	**5**
*Asparagus africanus *Lam.	4	3	6	3	6	4	3	4	5	3	3	5	1	4	54	**2**
*Dioscorea quartiniana A. Rich*.	5	5	5	1	2	2	4	2	3	1	6	4	6	3	49	**3**
*Pittosporum viridiflorum Sims*	1	1	3	2	3	6	6	5	1	4	1	1	2	1	37	**6**
*Hydnoria johannis Becc*.	2	4	2	6	5	5	1	1	4	2	2	3	3	5	45	**4**
*Withania somnifera (L.) Dun*.	6	6	4	5	4	3	5	6	2	6	4	6	5	6	68	**1**

### Medicinal plants used for purposes other than medicinal value

In Mana Angetu the majority of the inhabitants rely on wild plants for various purposes such as forage, medicine, firewood, charcoal making, construction and food. It was found that 99 species (43.04%) of medicinal plants have values other than their medicinal role. To assess the relative importance and to check the major impact on such plants direct matrix ranking was performed.

The result indicated that *Olea europaea *was ranked first followed by *Acacia tortolis*, *Cordia africana *and *Warburgia ugandensis *Sprague (Table 6). This indicates that the plants were known for their values other than their medicinal role and this could be associated directly with the cause of their depletion in the study area. It also indicates that special focus should be given for conservation of these plants since they are being widely exploited for purposes other than their medicinal value.

**Table 6:** Average score for direct matrix ranking of 7 medicinal plant species with different uses other than medicinal use.

No	**Use diversity**	*Acacia tortilis*	*Warburgia ugandensis*	*Euclea divinorum*	*Cordia africana*	*Croton macrostachyus*	*Olea europaea*	*Asparagus africanus*
1	Fire wood	5	5	3	3	4	5	5
2	Charcoal	5	1	0	2	2	3	3
3	Medicine	3	5	5	4	4	4	4
4	Building	3	4	2	5	3	5	3
5	Forage	3	1	2	1	1	1	2
6	Furniture	2	2	0	4	2	5	1
								
**Total**		**121**	**90**	**73**	**102**	**70**	**125**	**82**
**Rank**		**2**	**4**	**6**	**3**	**7**	**1**	**5**

Based on use criteria (5 = best; 4 = Very good; 3 = good; 2 = less used; 1 = least used and 0 = no value)

### Threats to medicinal plants and conservation practices

In Mana Angetu District various factors that were considered as main threats for medicinal plants were recorded by interviewing the informants. The major factors claimed were deforestation (90%), agricultural expansion (85%), fire (53%), overgrazing (15%), drought (12%) and trading charcoal and firewood (10%). Other research on threats to medicinal plants used by Kereyu pastoralists in Ethiopia [29] indicates similar investigation.

The effort to conserve medicinal plants in the district was observed to be very poor. Some traditional practitioners have started to conserve medicinal plants by cultivating at home gardens, though the effort was minimal. About 5.7 %, of the medicinal plants collected were reported as found cultivated at home gardens and these include plants like *Carica papaya *L., *Catha edulis *(Vahl) Forssk. ex Endl., *Coffea arabica*, *Jatropha curcas *L., *Prunus perisca *(L.) Batsch and *Euphorbia piscidermis *M. Gilbert. Traditional beliefs in the area also have their own unintentional role in conservation and sustainable utilization of medicinal plants.

Giving conservation priority for identified threatened medicinal plants, promoting *in-situ *and *ex-situ *conservation of medicinal plants in Mana Angetu area as well as supporting the district's Traditional Healers Association, by providing funds, land for cultivating medicinal plants and assisting their activities with professional guidance helps to conserve the fast eroding medicinal plants of the study area.

## Competing interests

The authors declare that they have no competing interests.

## Authors' contributions

All authors contributed equally during the field work, data analysis and preparation of the manuscript.

## Supplementary Material

Additional file 1 The additional file shows pictures reflecting some of the field data collection events. Click here for file

Additional file 2 Medicinal plants used for treatment of human and/or livestock diseases. The additional file lists plant species used to treat human and/or livestock ailments, and methods of preparation and application. Click here for file
