# Supplementary material for: An ethnobotanical study of medicinal plants in Mana Angetu District, southeastern Ethiopia
Source: J Ethnobiol Ethnomed. 2008 Apr 28;4:10. doi: 10.1186/1746-4269-4-10 (PMC2391147; doi:10.1186/1746-4269-4-10)
Supplement: Additional file 1 — The additional file shows pictures reflecting some of the field data collection events. [file 1746-4269-4-10-S1.pdf]

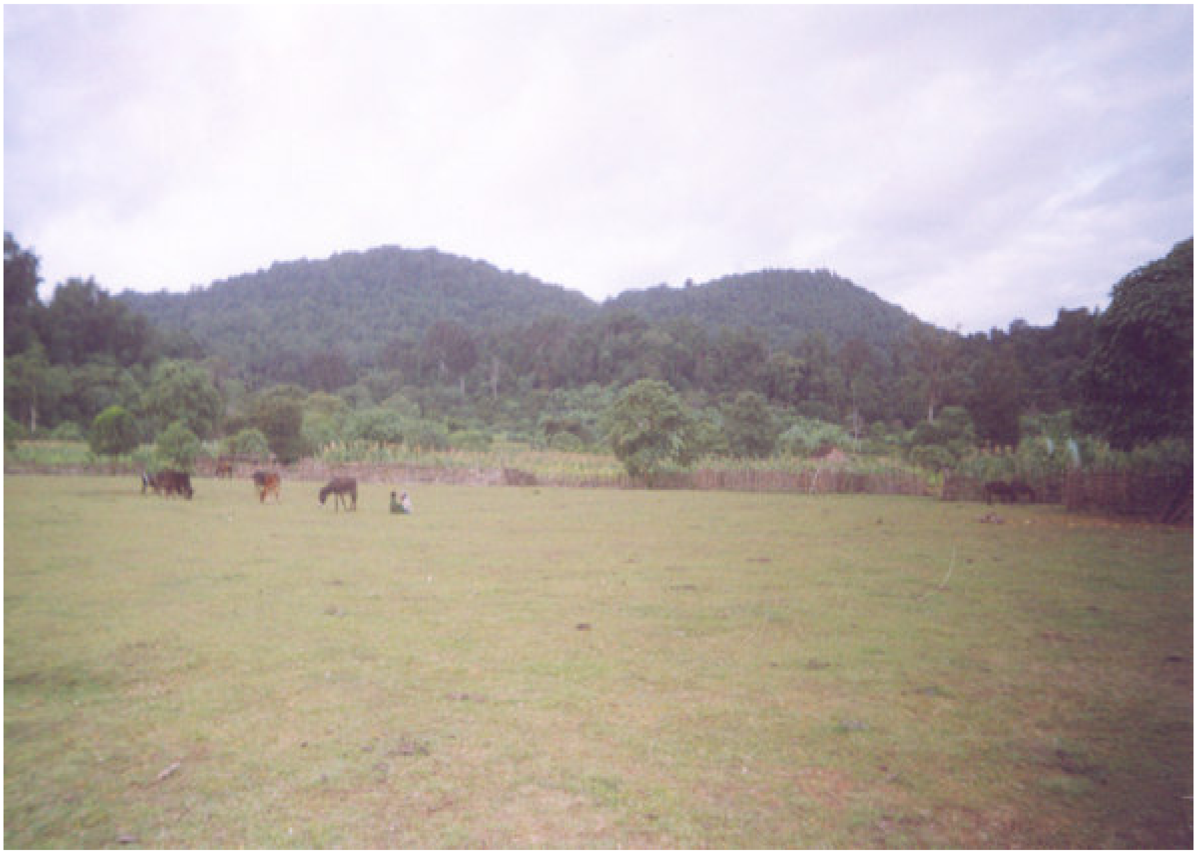

Partial view of the study area

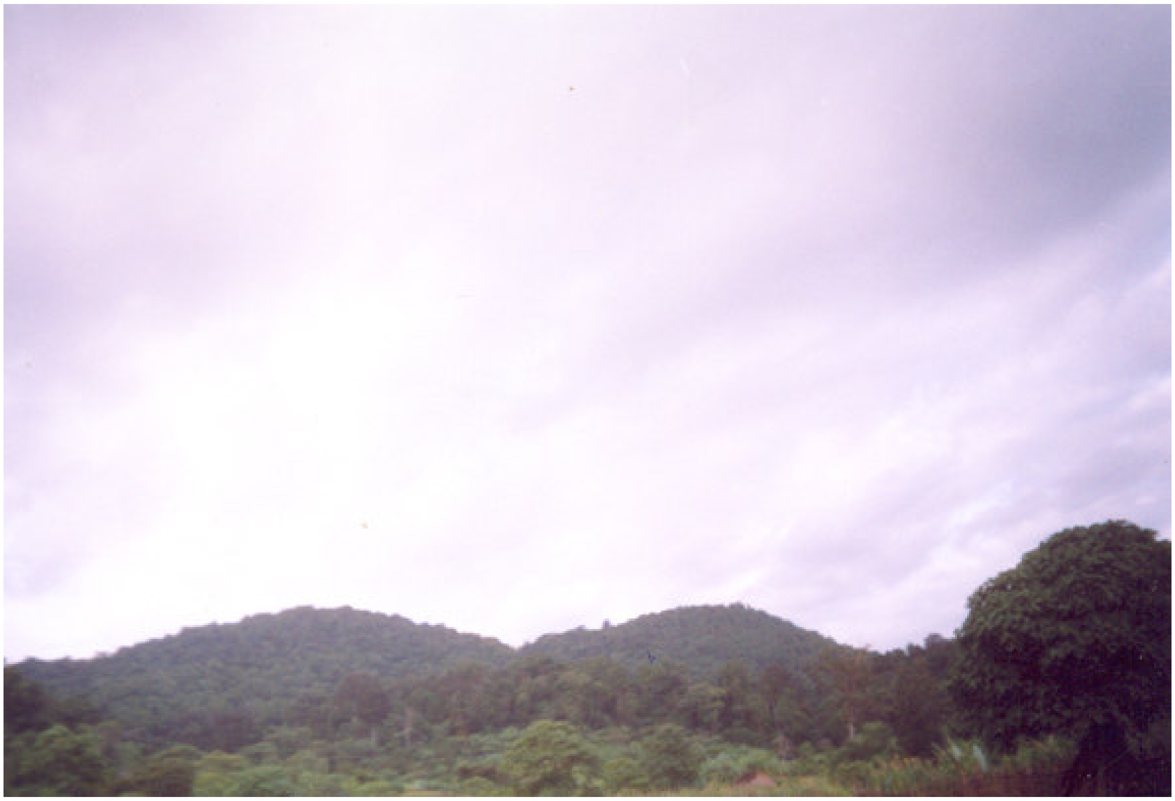

Partial view of the study area

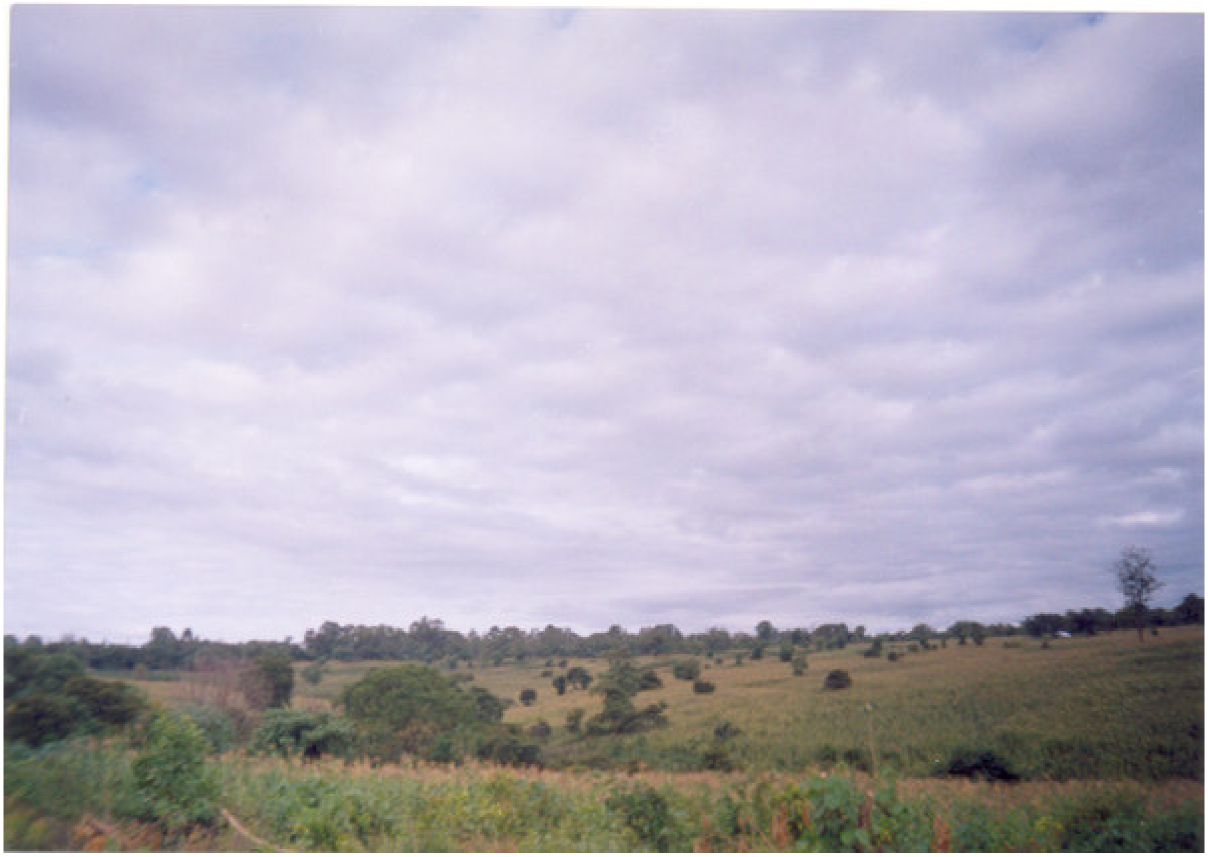

Partial view of the study area

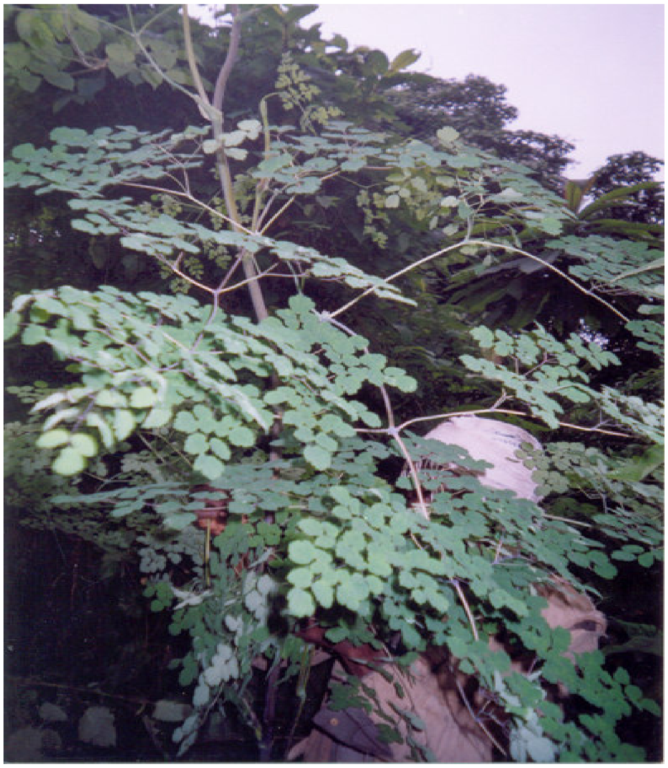

Thalictrum rhynchocarpum **Dill. & A. Rich.**

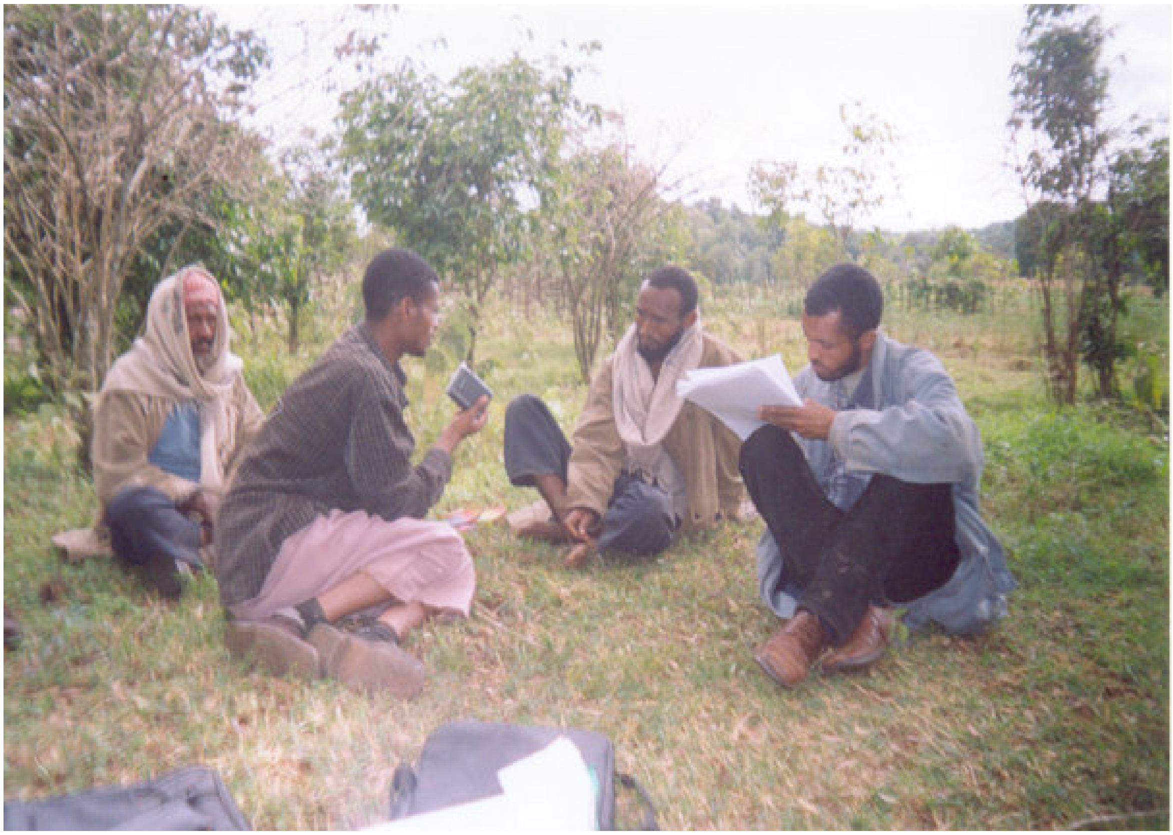

Interview

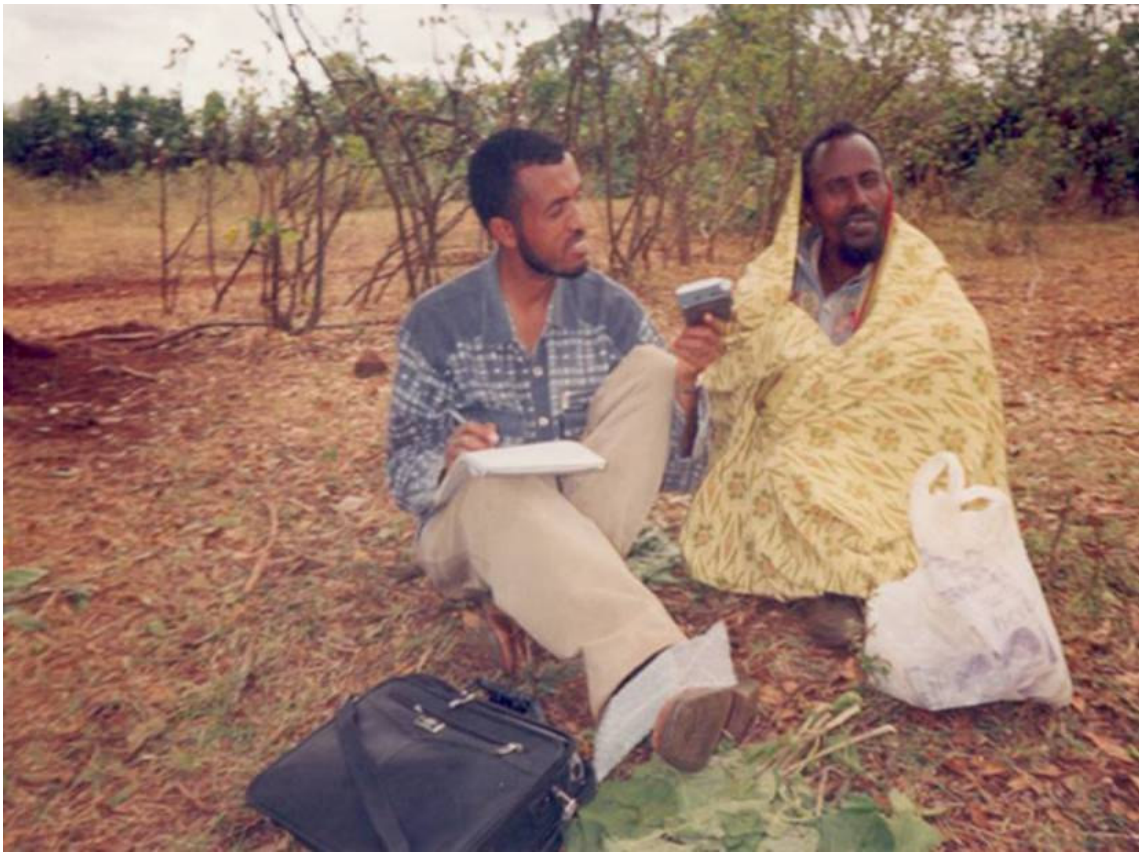

Interview

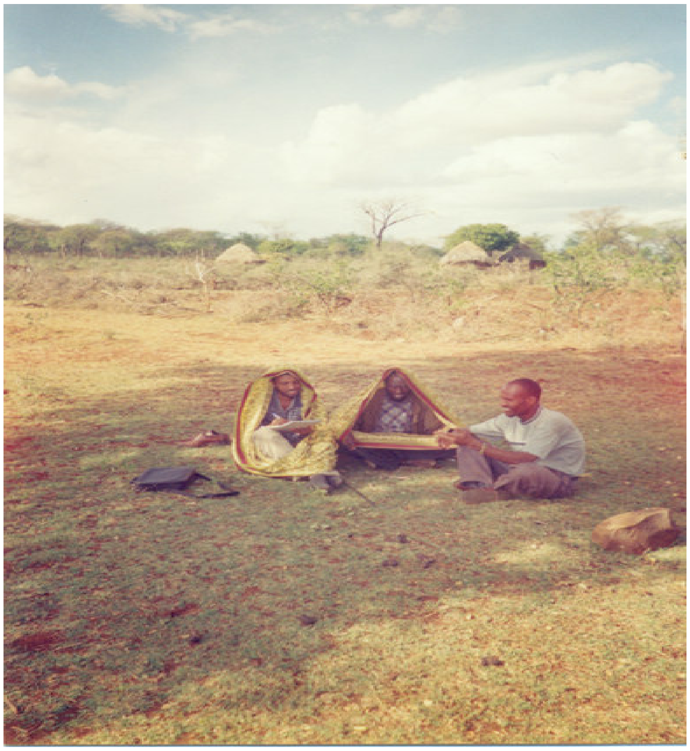

Interview

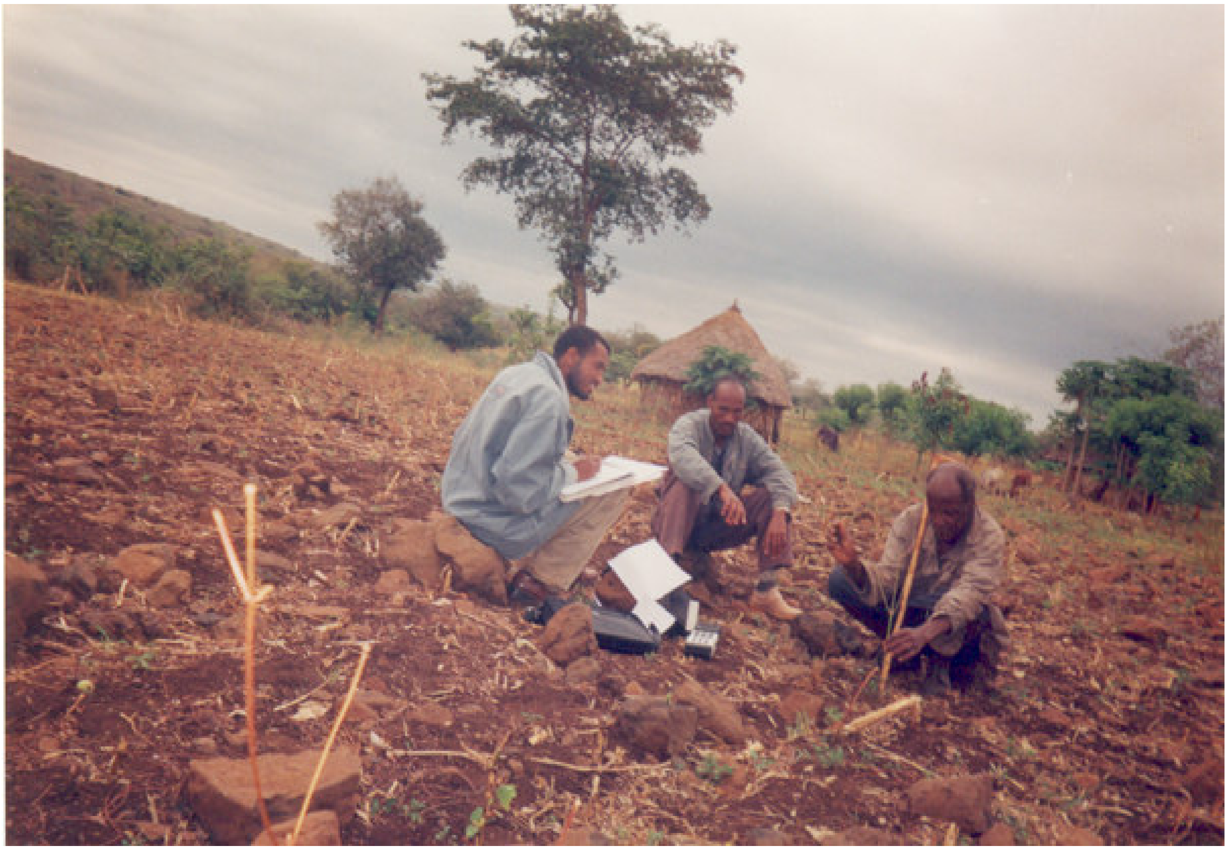

Interview

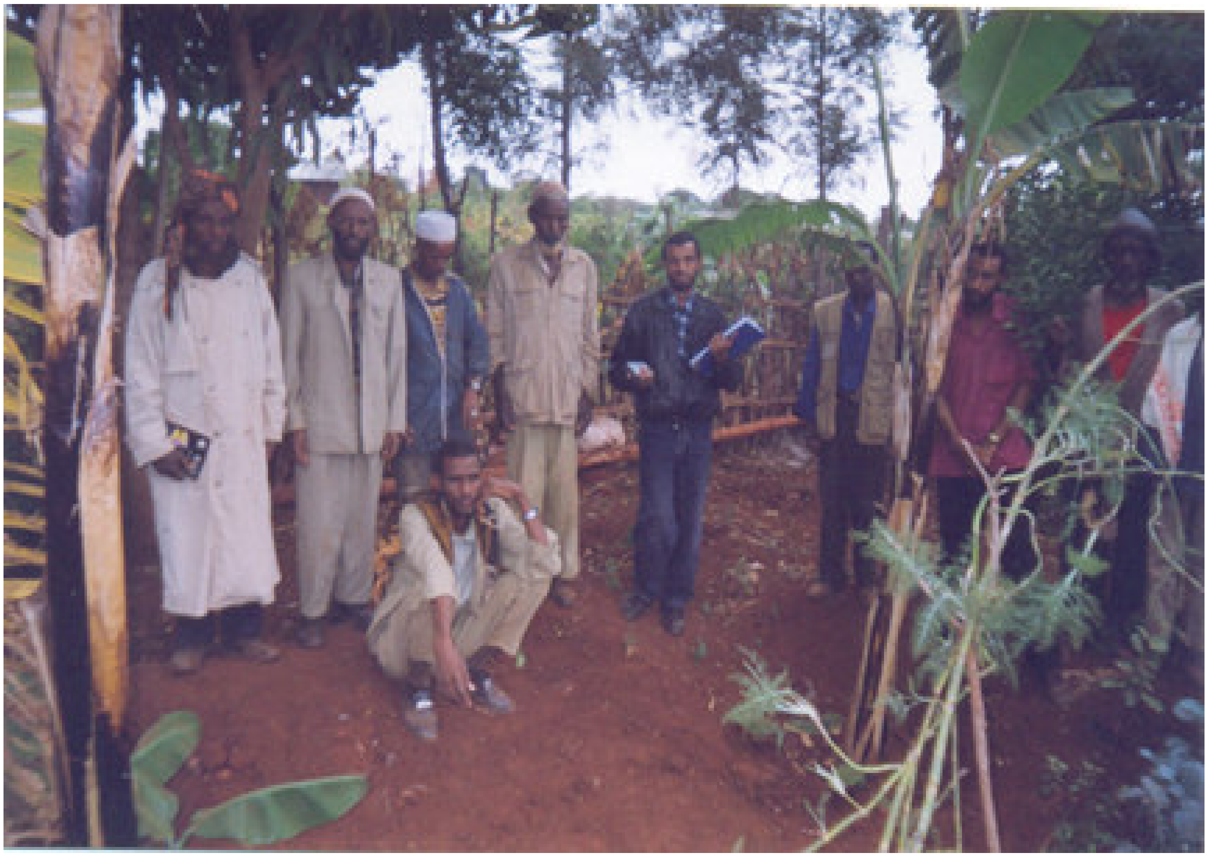

Key informants

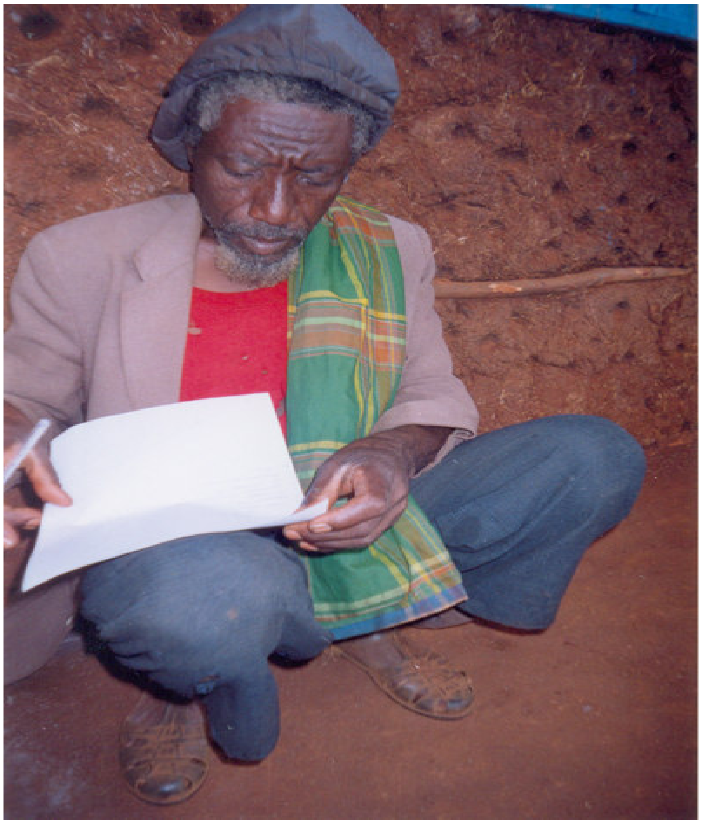

One of the informants

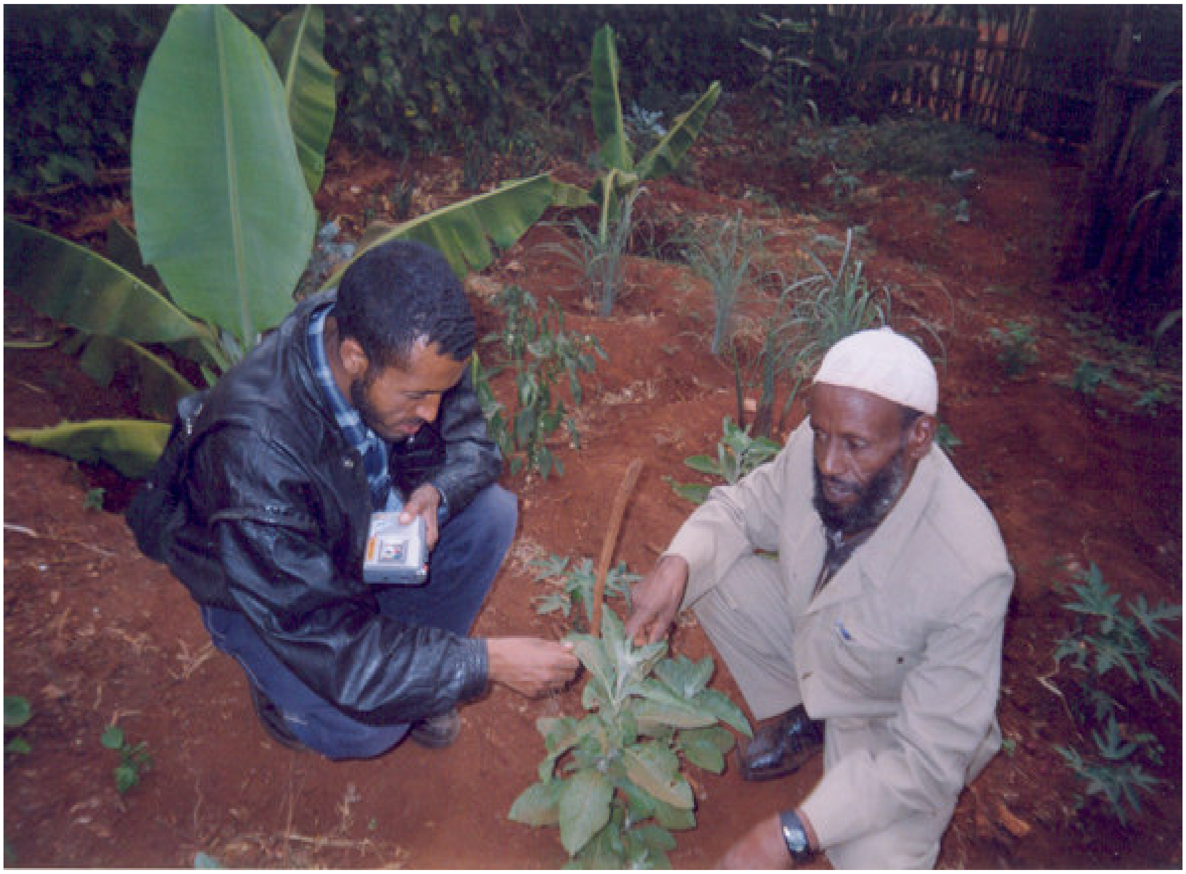

A well known traditional healer who is acting as the chair person of the practitioners' association demonstrating his medicinal plants planted in his home garden

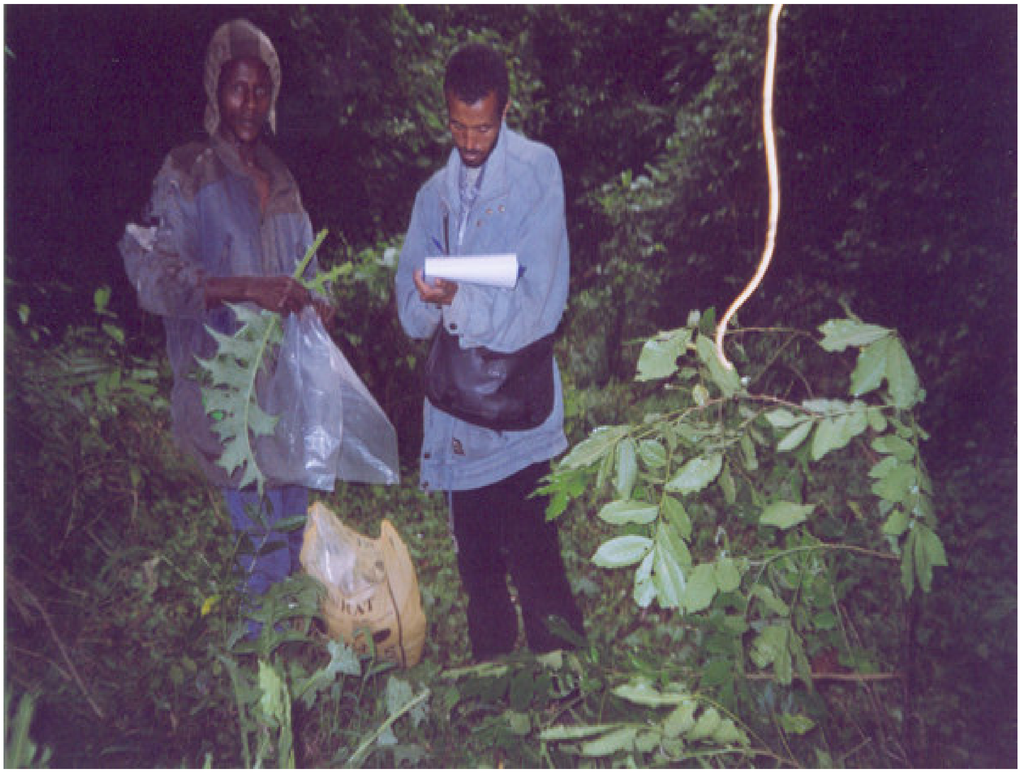

Medicinal plant collection from the field

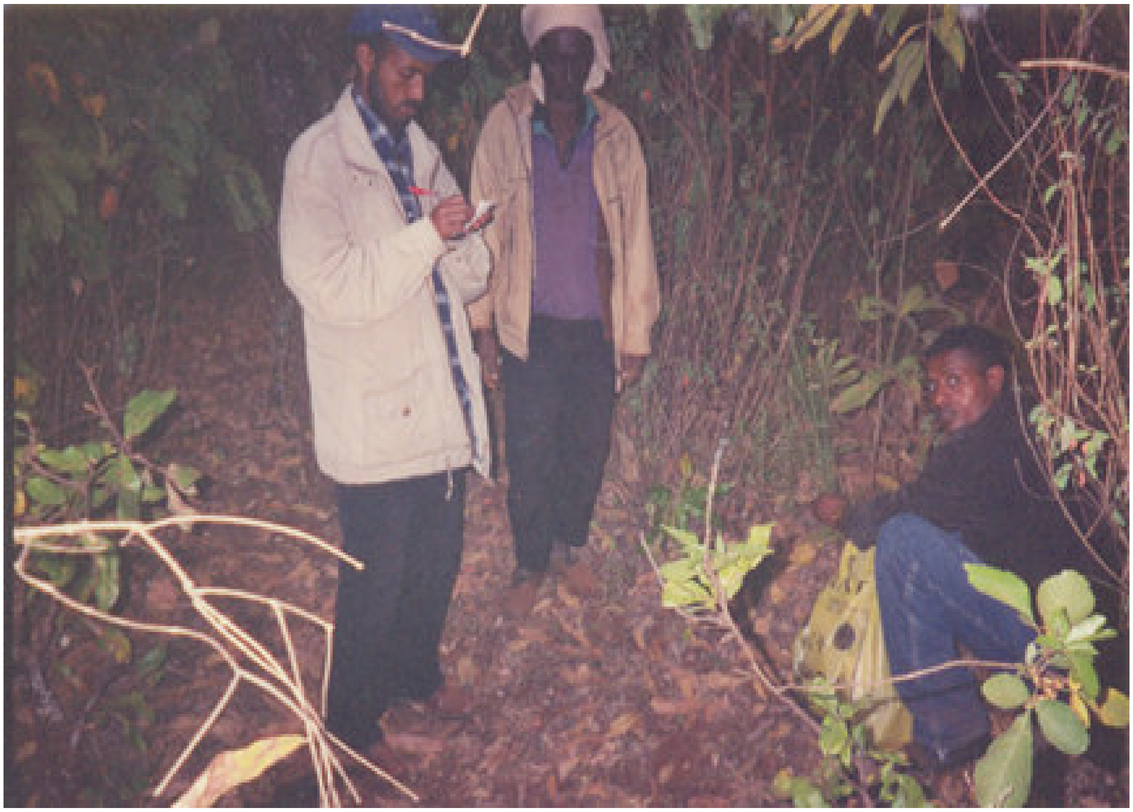

Medicinal plant collection from the field

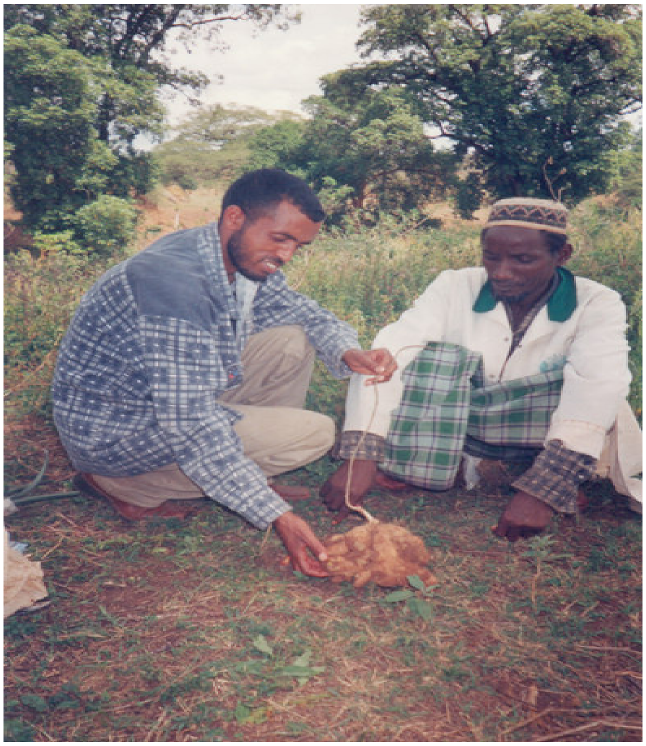

Medicinal plant collection from the field

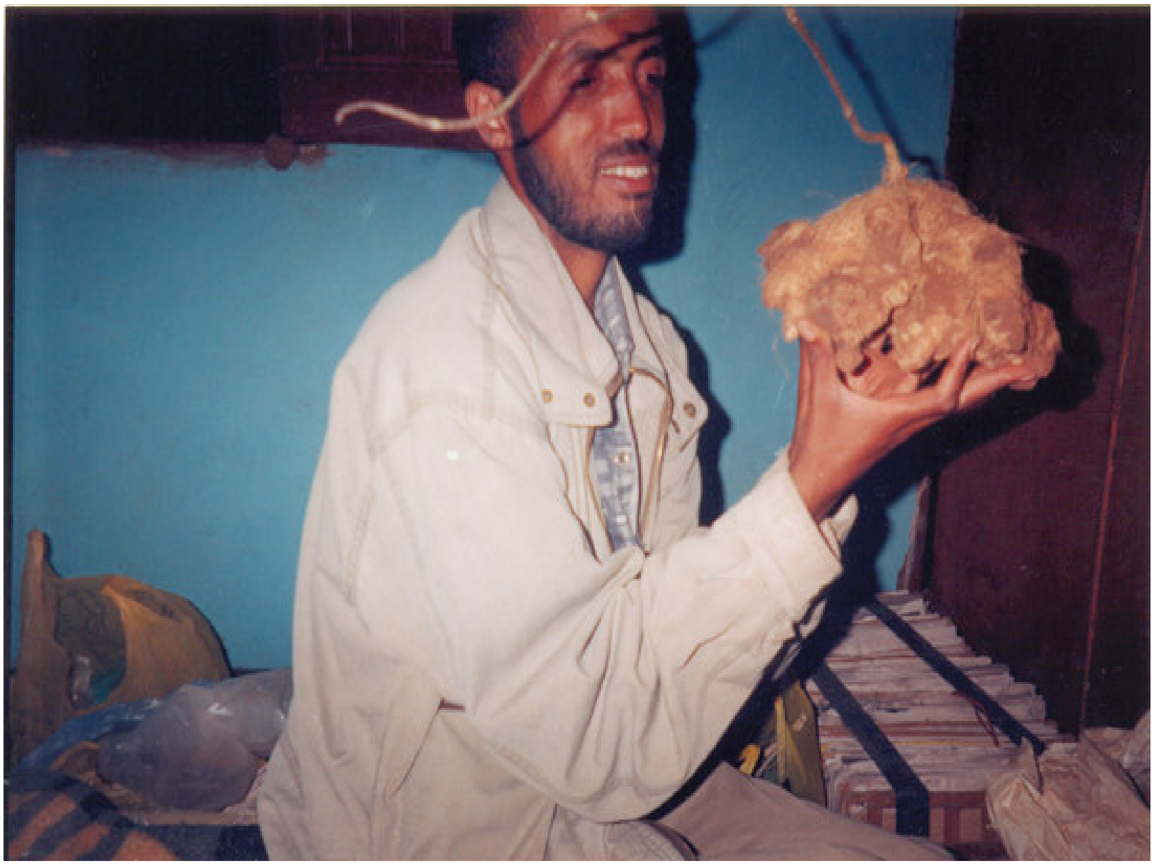

An impression immediately after pressing specimens

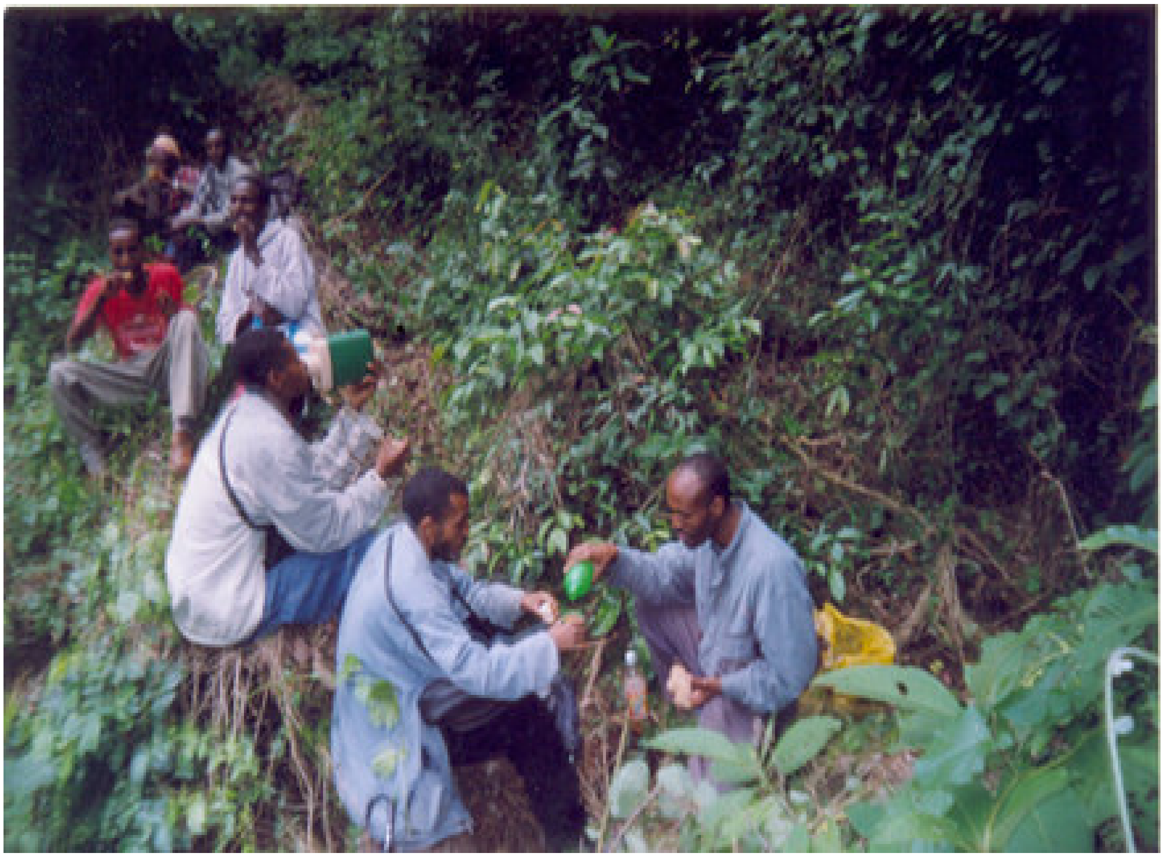

Lunch time
