# Supplementary material for: An ethnobotanical study of medicinal plants in Mana Angetu District, southeastern Ethiopia
Source: J Ethnobiol Ethnomed. 2008 Apr 28;4:10. doi: 10.1186/1746-4269-4-10 (PMC2391147; doi:10.1186/1746-4269-4-10)
Supplement: Additional file 2 — Medicinal plants used for treatment of human and/or livestock diseases. The additional file lists plant species used to treat human and/or livestock ailments, and methods of preparation and application. [file 1746-4269-4-10-S2.pdf]

Additional file 2. Medicinal plants used for treatment of human and /or livestock diseases

Scientific name	Family	Local name	Herb. Vouch.	Disease treated (local name)	L/H	Part used	Form used	MPAP
Abutilon mauritianum (Jacq.) Medic.	Malvaceae	Pelampul	EL154	Snake bite/ Boffa	H	R	F	CT
Acacia etbaica Schweinf.	Fabaceae	Hamaressa	EL167	Inability to walk properly /Dereba	L	R	F/D	Cdr
Acacia mellifera (Vahl *)* Benth.	Fabaceae	Bilala	EL230	Kidney/ Birbirti	H	R	D	CBdr.
Acacia senegal (L.) Willd.	Fabaceae	Sapansa	EL170	Evil spirit/ Ka falfala	H	S	F/D	CR
Acacia seyal Del.	Fabaceae	Watcho	EL158	Breast pain /Dhukuba harma	L	R	F/D	Cdr
Acacia tortilis (Forssk.) Hayne	Fabaceae	Dedecha/Kitkita	EL159	Problem of removing after birth/ Ka hobati	L	L	F	Cdr
Acalypha fruticosa Forssk.	Euphorbiaceae	Gurbi dag Hadoftu	EL232	Tonsilities/ Entil	H	R	F/D	Cdr
Acanthospermum hispidum DC.	Asteraceae	Qummuxxi	EL136	Sterility in females/ Mehaninet	H	R	F	CBdr.
Achyranthes aspera L.	Amaranthaceae	Jibir	EL104	Itching sore/ Aro	H	L	F	CBdr.
Acokanthera schimperi (A.DC.) Schweinf.	Apocynaceae	Kararo	EL100	Gonorrhoea/ Chobto	H	RB	F	CBdr
Actinopteris radiata (L.) P. Beauv.	Actinopteridaceae		EL187	Stabbing pain/ Woransa	H	L	F	CT
Aerangis brachycarpa (A. Rich.) Th. Dur. & Schinz	Orchidaceae	Muka kulubi	EL239	Hemorrhoids/ Qormade	H	L	F	CRrb
Ageratum conyzoides L.	Asteraceae		EL87	Gonorrhoea/ Chobto	H	R	F	CBdr.
Allophylus macrobotrys Gilg	Sapindaceae	Chobsa	EL117	Impotence /Dhirumalebi	H	R	D	Cdr
Aloe gilbertii Sebsebe & Brandham	Aloaceae	Hargessa	EL209	Liver case/ Hadhoftu	H	LA	F	CEJ
Aloe parvidens Gilbert & Sebsebe	Aloaceae	Hargessa	EL208	Stabbing pain/ Woransa	H	LA	F	Cdr
Aloe pubescens Reynolds	Aloaceae	Hargessa	EL207	Common cold/ Qufa	L	S	F	CR
				Dhukuba dugda	H	S	D	CBdr.
				TB /Sombe	H	S	D	Cdr

Additional file 2 (continued)

Scientific name	Family	Local name	Herb. Vouch.	Disease treated (local name)	L/H	Part used	Form used	MPAP
Aloe pubescens Reynolds	Aloaceae	Hargessa	EL207	Kidney /Birbirti	H	S	F	Cex
				Cold /Qilensa	H	S	F	Cex
Ampelocissus abyssinica (Hochst. ex A. Rich.) Planch.	Vitaceae	Teru	EL175	Black leg/Aba gorba	L	R	F/D	CBdr.
Anethum graveolens L.	Apiaceae	Kamona	EL29	Eye disease/ Dukuba ija	H	LA	F	CR
				Back pain/ Kale	H	L	F	Cdr
				Impotence/ Dhirumalebi	H	RLSF	F	CBdr.
				Weakness/ Dukubaone	H	LSB	F/D	CBdr.
Asparagus africanus Lam.	Asparagaceae	Argeg	EL189	Weakness/ Dukubaone	H	R	F	CT
Asparagus leptocladodius Chiov.	Asparagaceae	Seriti	EL190	Impotence/ Dhirumalebi	H	R	D	Cdr
				Liver case /Hadhoftu	H	R	F	Cdr
				Rabies/ Dukuba serie	H	R	F/D	CBdr.
				Depresssion /Gafla	H	L	F	CBdr.
Aspilia gillettii Wild	Asteraceae		EL113	Liver case/ Hadhoftu	H	RL	F/D	CBdr.
				Abnormal Menustration/ dhiga	H	L	D	CT
Asystasia guttata (Forssk.) Brummitt	Acanthaceae	Baquli	EL191	Diarrhoea/ Bassa	H	R	F/D	CR
				Evil spirit/Jinni	H	R	F	CR
Balanites aegyptica (L.) Del.	Balanitaceae	Badanna	EL147	Evil spirit/ Jinni	H	R	F/D	CR
Barleria argentea Balf. f.	Acanthaceae	Hokto	EL194	Diarrhoea /Albati	H	RL	F	Cdr
Barleria eranthemoides R. Br. ex C. B. Clarke	Acanthaceae	Shishi	EL193	Stabbing pain/ Woransa	H	R	D	CBdr.
				Cold/ Ka fatime	H	R	F	CR
				Taenia versicolor/ Barley	H	L	F	CRrb
Bersama abyssinica Fresen.	Melianthaceae	kilisa guracha	EL173	Ascariasis /Magga	H	L	F	CBdr.

Additional file 2 (continued)

Scientific name	Family	Local name	Herb. Vouch.	Disease treated (local name)	L/H	Part used	Form used	MPAP
Blyttia fruticulosum (Decne.) D.V. Field	Asclepiadaceae	Joshani	EL221	Vomit& diarrhoea/ Wantufa	H	SB	F	CBdr.
Boscia salicifolia Oliv.	Capparidaceae	Qalqalchi qalada	EL111	Diarrhoea /Tuma	L	RB	D	Cdr
Boscia senegalensis Lam. ex Poir.	Capparidaceae	Qalqalcha	EL182	Headache/ Bowo	H	RL	F	CT
Breonadia salicina (Vahl) Hepper and Wood	Rubiaceae	Erba	EL142	Intestinal worms/Maga	L	SB	F/D	CBdr.
Brucea antidysentrica J. F. Mill.	Simaroubaceae	kilisa adi	EL152	Gonorrhoea/ Chobto	H	L	F	CBdr.
				Evil spirit/ Jinni	H	L	F	CP
Caesalpinia volkensii Harms	Fabaceae	Sidika	EL233	Sterility in females/ Mehaninet	H	Se	D	Cdr
Calpurnia aurea (Ait.) Benth.	Fabaceae	Chekata	EL76	Amoebiasis /Dubera	H	S	F/D	CBdr.
				Swelling of nose in mule/ Chachabsa	L	S	F/D	CR
				Sore and depression/ Hinjira	L	L	F	CR
				Rabies /Dukuba serie	H	R	F/D	CPdr
				Diarrhoea/ Bassa	H	L	F/D	CBdr.
Capparis tomentosa Lam.	Capparidaceae	Gombor	EL121	Mental disorder/ Ka marata	H	R	F	CB
				Meningites/ Dhukubadhira	H	R	F	Cdr
Capsicum frutescens L.	Solanaceae	Mitmita	EL204	Diarrhoea /Tuma	L	F	D	Cdr
Caralluma peckii Bally	Asclepiadaceae	Hadama	EL118	Gonorrhoea/ Chobto	H	R	F/D	Cdr
Caralluma speciosa (N.E. Br.) N.E. Br.	Asclepiadaceae		EL132	Ascariasis/ Magga	H	R	F	CBdr
Carica papaya L.	Caricaceae	Papaya	EL40	Anaemia/ Helina diga	H	F	F/D	Cdr.
Carissa spinarum L.	Apocynaceae	Agamsa	EL16	Mental disorder/ Marata	H	R	F	Csn
				Evil spirit/Jinni	H	R	F	CR
				Evil eye/ Buda	H	R	F/D	CR
				Rabies/ Dukuba serie	H	RB	F/D	Cdr
				Rabies/ Dukuba serie	H	R	F/D	CBdr.

Additional file 2 (continued)

Scientific name	Family	Local name	Herb. Vouch.	Disease treated (local name)	L/H	Part used	Form used	MPAP
Carissa spinarum L.	Apocynaceae	Agamsa	EL16	Kidney /Birbirti	H	SB	F	CBdr.
				Skin infection /Beresa	H	R	F	CRbo
Cassytha filliformis L.	Lauraceae	Korsa buda	EL79	Evil eye / Buda	H	R	F/D	CR
Catha edulis (Vahl) Forssk. ex Endl.	Celasteraceae	Chati	EL26	Gonorrhoea/ Chobto	H	L	F	CBdr.
Caucanthus auriculatus (Radlk.) Niedenzu	Malphigiaceae	Alotu	EL186	Evil eye / Buda	H	RB	F	Cdr
Chenopodium ambroisoides L.	Chenopodiaceae	Ajo	EL4	Dandruff /Forofori	H	LSS	D	CR
Chionanthus mildbraedii (Gilg & Schellenb.) Stearn	Oleaceae	Gaggama	EL183	Inability of excretion/Dhukuba dira	H	RB	F	CBdr.
Cissampelos pareira L.	Menispermaceae	Baltoke	EL77	Evil eye/ Buda	L	R	F	Cdr/Cp
				Impotence/ Dhirumalebi	H	R	D	Cdr
				Kidney /Birbirti	H	RL	F/D	Cdr
				Amoebiasis/ Dubera	H	R	F	CBdr.
				Abnormal Menstruation/.Dhukuba dhiga	H	R	F/D	CBf
				Constipation/ Ka mara	H	R	F	CBdr.
				Diarrhoea /Tuma	H	R	F/D	Cdr
				Stabbing pain/ Woransa	H	R	F/D	CBdr.
Cissus populnea Guill. and Perr.	Vitaceae	Gangalto	EL115	Vomit& diarrhoea /Wantufa	H	R	F/D	CBdr.
Cissus quadrangularis L.	Vitaceae	Chopi	EL176	Gonorrhoea /Chobto	H	R	F	CBdr.
Citrus limon (L.) Burm.f.	Rutaceae	Lemon	EL155	Bleeding nose/ Nasir	H	FL	F/D	Cex
Citrus medica L.	Rutaceae	Tiringo	EL114	Cold /Kebena	H	L	F	Cb
				Swelling/Gofla	H	L	F/D	CT/Cdr
Clematis hirsuta Perr. and Guill.	Ranunculaceae	Fitti	EL18	Naqarssa	L	L	F	CT
Clematis simensis Fresen.	Ranunculaceae	Fitti	EL43	Naqarssa	L	R	F	CT
Clerodendrum alatum Gurke	Lamiaceae	Hawore	EL70	Mental disorder /Marata	H	R	F	CBdr.

Additional file 2 (continued)

Scientific name	Family	Local name	Herb. Vouch.	Disease treated (local name)	L/H	Part used	Form used	MPAP
Clerodendrum alatum Gurke	Lamiaceae	Hawore	EL70	Hepatitis /Dukuba tiru	H	R	F/D	CBdr.
Clerodendrum myricoides (Hochst.) Vatke	Lamiaceae	Marasisa	EL201	Mental disorder /Marata	H	RB	F	Csn
				General malaise /Mich	H	L	F	CR
				Cold /Qilensa	H	RB	F	Cdr
Clutia abyssinica Jaub. and Spach.	Euphorbiaceae		EL10	Skin infection/ Chito	H	L	F	CR
				Bloody diarrhoea/ Setea	H	R	F/D	CBdr.
Coccinia grandis (L.) Voigt	Cucurbitaceae	Gale	EL141	Kidney infection /Birbirti	H	R	F/D	CR
				Stabbing pain /Woransa	H	R	F/D	Cdr
Coffea arabica L.	Rubiaceae	Bunna	EL12	Kidney infection/ Birbirti	H	L	F	CBdr.
				Hepatitis /Dukuba tiru	H	S,Se	F	CBdr.
Colocassia esculenta (L.) Schott	Araceae	Gondire	EL196	Vomit& diarrhoea /Wantufa	H	L	F	CRrb
Combretum hereroense Schinz	Combretaceae	Hurufo	EL148	Sore /Sibiji	H	L	F	Crb
Commicarpus plumbagineus (Cav.) Standl.	Nyctaginaceae	Sifa	EL225	Stabbing pain /Woransa	H	R	D	CBdr.
Convolvulus siculus L.	Convolvulaceae	LeIbab	EL236	Diarrhoea /Tuma	H	L	F/D	CBdr
Cordia africana Lam.	Boraginaceae	Wodessa	EL56	Gastritis/ Agano	H	SB	F	ChSw
				Sore /Dhukuba mada	H	L	F	CT
				Sore/ Sibiji	H	L	F	CR
Crabbea velutena S. Moore	Acanthaceae		EL59	Gonorrhoea /Chobto	H	R	F	CBdr
Crambe hispanica L.	Brassicaceae	Fugul (Arabic)	EL92	Naqarssa	H	R	F	CBdr.
Crotalaria deserticola Bak. f.	Fabaceae		EL48	Naqarssa	H	R	D	CBdr

Additional file 2 (continued)

Scientific name	Family	Local name	Herb. Vouch.	Disease treated (local name)	L/H	Part used	Form used	MPAP
Crotalaria spinosa Hochst. ex Benth.	Fabaceae		EL161	Epilepsy in cattle/ Furtu	L	L	F	Cdr
Croton dichogamus Pax	Euphorbiaceae	Makuffa	EL101	Skin infection/Chito/Ikek	H	RB	F/D	CB
				Cold/ Qilensa	H	R	D	Csm
				Diarrhoea/Qirt bussa	L	R	D	Cdr
Croton macrostachyus Del.	Euphorbiaceae	Bakanissa	EL17	Swelling of nose in mule /Chachabsa	L	L	F/D	CR
				Epilepsy /Gagabdo	H	SB	F/D	Cdr.
				Epilepsy in cattle /Furtu	L	L	F/D	CO
				Hepatitis /Dukuba tiru	H	RB	D	CE
				Ascariasis /Maga	H	SBRB	F/D	Cdr
				Epilepsy in cattle /Furtu	L	L	F	Cex
				Gonorrhoea /Sure	H	R	D	CE
Croton schimperianus Muell. Arg.	Euphorbiaceae	Makafta	EL219	Evil spirit/ Jinni	H	L	F/D	Csm
				Cold /Qilensa	H	R	D	Csm
Cryptostegia grandifolia Roxb. ex R. Br.	Asclepiadaceae	Headache	EL131	Sore/ Sibiji	H	L	F/D	Crb
Cucumis dipsaceus Ehrenb. ex Spach	Cucurbitaceae	Kurera	EL140	Meningitis/ Dhukuba dhira	H	F	F	Cex
				Hemorrhoid/ Qormade	H	F	F	Cex
				Rabies/ Dukuba serie	H	F	F	Cex
Cucumis ficifolius A. Rich.	Cucurbitaceae	Hanchote	EL68	Rabies /Dukuba serie	H	R	F/D	CPdr
				Lung disease /Sombe	L	RF	F	Cdr
				Gonorrhoea /Chobto	H	RF	F/D	Cdr
				Jaundice /Alati	H	R	F/D	CBdr.
Cucurbita pepo L.	Cucurbitaceae	Buke	EL96	Epilepsy in cattle /Furtu	L	L	F	Cdr
Cussonia holstii Harms ex Engl.	Araliaceae	Bubiftu	EL99	Headache and fever/ Qora	H	L	F	CR
Cynanchum abyssinicum Decne.	Asclepiadaceae	Muka Aro	EL6	Sore Dukuba /Aro	H	L	F/D	Crb
Cynanchum clavidens N.E. Br.	Asclepiadaceae	Halala	EL81	Tenia versicolour/ Barley	H	L	F	Crb

Additional file 2 (continued)

Scientific name	Family	Local name	Herb. Vouch.	Disease treated (local name)	L/H	Part used	Form used	MPAP
Cynoglossum amplifolium Hochst. ex DC.	Boraginaceae	Karchaba	EL75	General malaise /Mich	H	L	F	CR
Cynoglossum coerulem Hochst. DC.	Boraginaceae	Matare	EL66	General malaise/ Mich	H	L	F/D	CR
				Impotence /Dhirumalebi	H	R	D	Crd
				Eye disease /Dukuba ija	H	L	F	Cex
Cyphostemma cyphopetalum (Fresen.) Descoings ex Wild & Drum.	Vitaceae	Likime	EL3	Diarrhoea /Tuma	L	R	F	Cdr
				Coughing only in Horse and Donkey /Killis	L	R	F/D	CR ex
				Inability to walk properly /Dereba	L	R	F	Cdr
Cyphostemma dembianense (Chiov.) Vollesen	Vitaceae	Namignata	EL177	Sore /Naffa Goggoysa	H	R	F/D	Cdr /P
Datura stramonium L.	Solanaceae	Bengi	EL241	Stabbing pain/ Qilensa	H	RF	D	CR
Desmodium velutinum (Willd.) DC.	Fabaceae	Hidda Diga	EL11	Intestinal parasites /Gara muru	H	R	F	CBdr
Dichrocephala chrysanthemifolia (Bl.) DC.	Asteraceae	Gurbii	EL88	Gonorrhoea/ Chobto	H	R	F	Cdr
Dioscorea quartiniana A. Rich.	Dioscoreaceae	Gishu	EL108	Vomit & diarrhoea /Wantufa	H	R	F/D	CT/Cdr
				Kidney infection /Birbirti	L	R	F/D	Cdr
				Side pain/ Woransa	H	R	F	CBdr.
Dodonaea angustifolia L. f.	Sapindaceae	Kitkitta	EL20	Hemorrhoids/ Qormade	H	RB	F/D	CBdr
Dorstenia foetida (Forssk.) Schweinf.	Moraceae	Serbi	EL138	Skin infection /Beresa/	H	R	F	CRrb
Dyschoriste multicaulis Lindau	Acanthaceae	Qorsa loo	EL198	Diarrhoea/ Tuma	L	L	F/D	Cdr

Additional file 2 (continued)

Scientific name	Family	Local name	Herb. Vouch.	Disease treated (local name)	L/H	Part used	Form used	MPAP
Dyschoriste multicaulis Lindau	Acanthaceae	Qorsa loo	EL198	Diarrhoea /Tuma	L	SB	F/D	Cdr
Echinops amplexicaulis Oliv.	Asteraceae	Kore Hare	EL21	Hepatitis /Wansimbira	H	R	F/D	CBdr
Erianthemum dregei (Eckl and Zeyh.) V. Tiegh.	Loranthaceae	Derto	EL41	Evil spirit /Gini	H	SL	F/D	CB
				Naqarssa	L	SL	F/D	CT
				Epilepsy in cattle /Merto	L	RSL	F/D	Cdr
				Sore /Sibiji	H	L	F	CRrb
Erythrina brucei Schwinef.	Fabaceae	Wolena	EL247	Problem of after birth/ Ka hobati	H	R	F	CBdr.
Erythrochlamys spectabilis Gurke	Lamiaceae	Qorsa adi michi	EL210	General malaise/ Mich	H	L	F	CRrb
				Tenia versicolor/ Barley	H	L	F	Crb
Eucalyptus saligna Sm.	Myrtaceae	Bahir Zaf	EL89	Stabbing pain/ Qilensa/	H	L	F	CB
Euclea divinorum Hiern	Ebenaceae	Meiesa	EL14	Gonorrhoea /Chobto	H	R	F/D	CR
				Kidney infection /Birbirti	H	SB	F	CE
				Hepatitis/ Dukuba tiru	H	RB	D	CE
Euphorbia dumalis S. Carter	Euphorbiaceae	Dargu adi	EL35	Liver case /Hadhoftu	H	R	F	Cdr
Euphorbia heterophylla L.	Euphorbiaceae	Ananole	EL218	Dandruff /Fororforii	H	S	F	Cex
Euphorbia lophiosperma S. Carter	Euphorbiaceae	Kelkelcha alati	EL231	Evil eye/ Buda	H	R	F/D	CR
Euphorbia piscidermis M. Gilbert	Euphorbiaceae	Kolkol	EL103	Gonorrhoea /Chobto	H	RS	F	CBdr.
				Evil spirit /Ka falfala/	H	S	F/D	CBdr.
				Hemorrhoids /Qormade/	H	S	F	CR
				Leprosy /Jusana	H	R	F	CE
Euphorbia schimperiana Scheele	Euphorbiaceae	Robe	EL36	Gonorrhoea/ Chobto	H	R	F/D	CBdr.
Faurea speciosa Welw.	Proteaceae	Bobeya	EL143	Skin infection/Ikek/ Chito	H	RB	F/D	Crb

Additional file 2 (continued)

Scientific name	Family	Local name	Herb. Vouch.	Disease treated (local name)	L/H	Part used	Form used	MPAP
Garcinia livingstonei T. Anders	Clusiaceae	Abuqurto	EL27	Hepatitis /Dukuba tiru	H	R	F/D	CBdr.
				Rabies /Dukuba serie	H	R	F/D	CPdr
				Urinary tract problem /Dukuba Finchani	H	R	F/D	Cdr
Gardenia ternifolia Schumach. & Thonn.	Rubiaceae	Gambella	EL248	Headache /Bowo	H	R	F/D	Cdr
				Pain of chest /Ka Qoma	H	R	D	CBdr.
Gloriosa superba L.	Colchicaceae		EL235	Epilepsy/ Gagabdo	H	R	F/D	CBdr
Gnidia stenophylla Gilg	Thymaelaceae	Harmala Tiqishu	EL7	Inability to walk properly /Dereba	L	R	F/D	Cdr
				Cold /Qilensa	H	R	F/D	Cdr
				Intestinal parasites /Gara muru	H	R	F/D	CBdr.
				Hemorrhoids /Qormade	H	R	F/D	Crb
				Gonorrhoea/ Chobto	H	R	F/D	CBdr.
Gomphocarpus fruticosus (L.) Ait. F	Asclepiadaceae	Dibe galla	EL133	Sore Dhukuba/ mada	H	L	F/D	Crb
Gomphocarpus integer (N.E. Br.) Bullock	Asclepiadaceae	Harmala	EL222	Gastritis /Agano	H	RL	F	Cdr
				Sore /Sibiji	H	L	F/D	CT
				Gonorrhoea/ Chobto	H	R	F/D	CE
				Hemorrhoids/ Qormade	H	RL	F	CR
				Kidney /Birbirti	H	R	F/D	CBdr.
				Gonorrhoea/ Chobto	H	R	F	CE
Gossypium arboreum L.	Malvaceae	Gibir	EL25	Skin infection/Ikek/ Chito	H	L	F	Crb
Grewia ferruginea Hochst. ex A. Rich	Tiliaceae	Hinshilo	EL217	Constipation /Ka mara	H	RL	F	Cdr
				Diarrhoea /Tuma	L	SB	F/D	Cdr
Harrisonia abyssinica Oliv.	Simaroubaceae	Tebenai	EL95	Inability to walk properly /Dereba	L	RL	D	Cdr
Heteromorpha arborescens (Spreng.) Cham. & Schltdl.	Apiaceae	Alhanka	EL30	Agressiveness in cows /Dhoitu	L	L	F	Cex
Hibiscus luduwigii Eckl. and Zeyh.	Malvaceae	Bulanbula	EL179	Diarrhoea /Bassa	H	R	F/D	Cdr

Additional file 2 (continued)

Scientific name	Family	Local name	Herb. Vouch.	Disease treated (local name)	L/H	Part used	Form used	MPAP
Hydnoria johannis Becc.	Hydnoraceae	Thuqa	EL78	Swelling of facial part/ Bofe	L	R	F	Cdr
Indigofera amorphoides Jaub. and Spach	Fabaceae	Gurbi adi	EL162	Lung disease /Sombe	H	R	F	CBdr
Indigofera dauensis Gillett	Fabaceae	Korsa Birti	EL128	Evil eye /Buda	H	R	F/D	CT
Indigofera spicata Forssk.	Fabaceae	Gurbi	EL166	Tenia versicolor /Barley	H	R	F/D	CR
				Diarrhoea/ Bassa	H	R	F	CBdr.
				Constipation /Mara	H	SB	F	Cdr
				Abnormal menstruation /Dhukuba dhiga	H	R	F/D	CBdr.
Inula decipiens E. A. Bruce	Asteraceae	Tamboborofa	EL135	Sore/ Sibiji	H	R	F	Crb
Inula paniculata (Klatt) Burtt-Davy	Asteraceae	Kora	EL51	Urinary tract problem / Finchani	H	R	F/D	Cdr
Jasminum schimperi Vatke	Oleaceae	Ilu	EL1	Evil spirit/ Gini	H	R	D	CR
Jatropha curcas L.	Euphorbiaceae	Abatal buluk	EL34	Headache/ Bowo	H	Se	F	CR
Justicia diclipteroides Lindau	Acanthaceae		EL60	Sore /Dhukuba mada	H	L	D	CR
Justicia odora (Forssk.) Lam.	Acanthaceae	Loketo	EL237	Depresssion/ Gafla	H	R	F/D	CBdr.
Kalanchoe laciniata (L.) DC.	Crassulaceae	Kontukature	EL139	Tenia versicolor /Barley	H	L	F	CR
				Eye disease/ Dukuba ija	H	L	F/D	Cex
Kalanchoe petitiana A. Rich.	Crassulaceae	Hanchura	EL94	Intestinal parasites /Gara muru	H	R	D	Cdr
Kanahia carlsbergiana Field et al.	Asclepiadaceae	Kertassa	EL82	Sore /Sibiji	H	L	D	Cpa
Kleinia abyssinica (A. Rich) A. Berger	Asteraceae	Burka	EL84	Vomit& diarrhoea /Wantufa	H	R	F/D	CBdr.
Kleinia squarrosa Cufod.	Asteraceae	Luqo	EL229	Cold /Kebena	H	R	D	Cb
Lagenaria siceraria (Molina) Standl.	Cucurbitaceae	Buke	EL28	Evil spirit /JinniGini	H	F	F	CT

Additional file 2 (continued)

Scientific name	Family	Local name	Herb. Vouch.	Disease treated (local name)	L/H	Part used	Form used	MPAP
Laggera crispata (Vahl) Hepper and Wood	Asteraceae	Dedeho	EL134	Abnormal menstruation/.Dhukuba dhiga	H	L	F	CT
Lannea schimperi (A. Rich.) Engl.	Anacardiaceae	Enxxilif	EL13	Skin infection /Chito	H	L	D	Crb
Lantana camara L.	Verbenaceae		EL200	Skin infection/Chito/Ikek	H	L	F	CRrb
				Gonorrhoea /Sure	H	R	F/D	CBdr.
				Evil eye /Buda	L	R	F	Cdr/Cp
Lantana trifolia L.	Verbenaceae	Medandubra	EL64	Sore /Mada	H	R	D	CP
				General malaise /Mich	H	L	F	CR
				Gonorrhoea/ Sure	H	R	F/D	CBdr.
				Evil eye /Buda	L	R	F	Cdr/Cp
Lantana viburnoides (Forssk.) Vahl	Verbenaceae	Medandubra	EL67	Jaundice/ Shinbira	H	R	D	CR
				Evil eye/ Buda	H	RL	F/D	CBdr.
Launea intybacea (Jacq.) Beauv.	Asteraceae	Lalessa	EL52	Impotence/ Dhirumalebi	H	R	F/D	CBdr.
Lepidotrichillia volkensii (Gurke) Ler.	Meliaceae	Sakero	EL249	Kidney /Birbirti	H	Se	F	Cex
Lippia javanica (Burm. f.) Spreng.	Verbenaceae	Sukahi	EL65	Diarrhoea /Albati	H	L	F	Cdr
				Jaundice /Alati	H	RL	D	CR
				Kidney disorder /Kulalit	H	L	D	Cdr
				Evil eye /Buda/	H	RL	D	Cdr
Maesa lanceolata Forssk.	Myrsinaceae	Dhunda/Abeyi	EL42	Skin infection/Chito/Ikek	L	Se	D	CPa
Maytenus arbutifolia (A. Rich.) Wilczek	Celasteraceae	Kombolcha	EL47	Wound/Madda/Dhita	L	L	F	CT
Maytenus senegalensis (Lam.) Exell	Celasteraceae	Kombolcha	EL45	Evil eye /Buda/	H	L	F	Csm
Melia azedarach L.	Meliaceae	Lucimia	EL91	Ascariasis /Maga	H	L	D	Cdr
Microglossa pyrifolia (Lam.) Kuntze	Asteraceae	Halalchu	EL144	Tenia versicolor /Barley	H	L	F	Crb

Additional file 2 (continued)

Scientific name	Family	Local name	Herb. Vouch.	Disease treated (local name)	L/H	Part used	Form used	MPAP
Mimusops kummel A. DC.	Sapotaceae	Qolati	EL151	Sterility in females /Mehaninet	H	SB	F	Cdr.
				Womb disorder /Hormata berra	H	RS	D	Cdr.
Myrsine africana L.	Myrsinaceae	Kechemsa	EL49	Skin infection /Chito	H	L	F	CRrb
Nicotiana tabacum L.	Solanaceae	Tambo	EL246	Epilepsy /Gagabdo	H	R	D	Cdr.
				Epilepsy in cattle/ Furtu	L	L	F	Cdr
Ocimum gratissimum L.	Lamiaceae	Dama Kessie	EL71	General malaise /Mich	H	L	F	Cdr.
Ocimum gratissimum L.	Lamiaceae	Dama Kessie	EL72	Eye disease/ Dukuba ija	H	L	F	Cex
Ocimum spicatum Defl.	Lamiaceae	Gurbi hare	EL214	Tenia versicolor/ Barley	H	L	F	CRrb
Ocimum urticifolium Roth	Lamiaceae	Korsa michi	EL69	General malaise/ Mich	H	L	F/D	CR
Olea europaea L. subsp. *Cuspidata* (Wall. ex G.Don)	Oleaceae	Ejersa	EL19	Leprosy /Jusana	H	RS	F	Distilation
				Mental disorder /Merata	H	L	F/D	CBdr.
				Urinary tract problem/ Finchani	H	L	F	Cdr
				Stabbing pain/ Woransa	H	L	F/D	CBdr.
Olinia rochetiana A. Juss.	Oliniaceae	Guna	EL102	Stabbing pain /Woransa	H	L	F/D	CBdr.
Ormocarpum trichocarpum (Taub.) Engl.	Fabaceae	Bute	EL125	Swelling of legs /Kurkassa	L	R	F/D	Cdr
Osyridocarpus schimperanus (A. Rich.) A. DC.	Santalaceae	Muca guracha	EL122	Jaundice /Alati	H	L	F	Cdr
Osyris quadripartita Decn.	Santalaceae	Wato	EL211	Pus from an ear /Dhukba guraa	H	RB	D	CR
Ozoroa insignis Del.	Anacardiaceae	Dheri	EL180	Gonorrhoea /Chobto	H	L	F/D	CBdr.
Pentanopsis fragrans Rendle	Rubiaceae	Kurso	EL216	Snakebite /Buti	H	RL	F	CRrb
				Gastritis /Agano	H	L	F/D	CBdr
Pentas lanceolata (Forssk.) Defl.	Rubiaceae	Dhumuga	EL213	Stabbing pain/ Woransa/	H	L	F/D	CBdr.

Additional file 2 (continued)

Scientific name	Family	Local name	Herb. Vouch.	Disease treated (local name)	L/H	Part used	Form used	MPAP
Periploca linearifolia Quart. -Dill. & A. Rich.	Asclepiadaceae	Sarkutal	EL83	Sore /Boffa	L	SL	F/D	CT
Phyllanthus sepialis Muell. Arg.	Euphorbiaceae	Sumalfer	EL2	Tooth ache /Dhukuba Hilkani	H	R	F	CRch
Phytolacca dodecandra L'.Herit.	Phytolacaceae	Handode	EL243	Gonorrhoea/ Chobto	H	R	F/D	CBdr.
Pittosporum viridiflorum Sims	Pittosporaceae	Ara	EL212	Stabbing pain/ Woransa	H	SB	F/D	Cdr.
Plumbago zeylanica L.	Plumbaginaceae	Digagi	EL58	Kidney /Birbirti	H	R	F	CBdr.
Podocarpus falcatus (Thunb.) Mirb.	Podocarpaceae	Birbirsa	EL197	Naqarssa	H	Rr	D	CP
				Womb disorder/ Lo'o	H	R	D	CBdr.
Portulaca oleracea L.	Portulacaceae	Doma	EL123	Nakarssa	H	R	F	CBdr.
Premna schimperi Engl.	Lamiaceae	Urgessa	EL202	Fire accident/ Ibida Nama Gubi	H	L	F	CPa
Prunus perisca (L.) Batsch	Rosaceae	Kok	EL32	Constipation and fever/ Oogera	H	L	F/D	Cdr
Psidium guagava L.	Myrtaceae	Zeyituna	EL38	Hepatitis /Wansimbira	H	Se	D	CBdr
Psychotria kirkii Hiern	Rubiaceae	Muka bofa	EL168	Snake bite /Bofa	H	RL	D	Cdr
Psychotria orophila Petit	Rubiaceae	Ulaga	EL15	General malaise /Mich	H	L	F	CRrb
				Jaundice /Alati	H	RL	F/D	Cdr
				Eye disease /Dukuba ija	H	L	F	Cex
Psydrax schimperiana (A. Rich.) Bridson	Rubiaceae	Gallo	EL165	TB /Sombe	H	R	F/D	CBdr.
Pterocephalus frutescesns Hochst. ex A. Rich.	Dipsacaceae		EL116	General malaise/ Mich	H	L	F	CR
Pterolobium stellatum (Forssk.) Brenan	Fabaceae	Kejima	EL46	Sore/ Sibiji	H	L	F	Cdr
				Stabbing pain /Woransa	H	R	F/D	CBdr.
Pyrenacantha malvifolia Engl.	Icacinaceae	Buri	EL106	Snakebite /Buti	H	R	F/D	CT

Additional file 2 (continued)

Scientific name	Family	Local name	Herb. Vouch.	Disease treated (local name)	L/H	Part used	Form used	MPAP
Rhoicissus tridentata (L. f.) Wild. & Drummond	Vitaceae		EL178	Abnormal menstruation/.Dhukuba dhiga	H	RS	F	CBdr.
Rhus natalensis Krauss	Anacardiaceae		EL120	Abnormal menstruation/.Dhukuba dhiga	H	RB	D	Cdr
Rhynchosia densiflora (Roth) DC.	Fabaceae		EL54	Vomit and Diarrhoea /Gudiftu/Wanejole	H	R	F	CR
Rhynchosia elegans A. Rich.	Fabaceae	Shabee	EL164	Jaundice /Alati/	H	R	F/D	Cdr
Rhyncosia ferruginea A. Rich.	Fabaceae		EL55	Evil eye/ Buda	L	RL	F	Cdr
Ricinus communis L.	Euphorbiaceae		EL33	Gonorrhoea /Chobto	H	R	F	Cex
Rubia cordifolia L.	Rubiaceae	Lalessa	EL53	Impotence/ Dhirumalebi	H	R	D	Cdr
				Pus from an ear/ Dhukba guraa	H	F	F	Cex
				Gonorrhoea/ Chobto	H	R	F/D	CBdr.
				General malaise/ Mich	H	L	F	Cdr
Rubus steudneri Schweinf.	Rosaceae	Goora	EL242	Headache/ Bowo	H	S	F	Cex
				Gonorrhoea /Chobto	H	L	F/D	CBdr.
Rumex nepalensis Spreng.	Polygonaceae	Shabee	EL24	Diarrhoea/ Bassa	H	R	F	CR
				Naqarssa	H	R	D	Cdr
Ruta chalepensis L.	Rutaceae	Siliti	EL37	Common cold /Qufa	H	L	F/D	CBdr
Sansevieria ehrenbergii Schweinf. ex Bak.	Dracenaceae	Muka koricha	EL238	Swelling/Ebach/Gofla	H	RB	F/D	CT
Satureja abyssinica (Benth.) Briq.	Lamiaceae	Korsa qufa	EL61	Common cold/ Qufa	H	L	F	CBdr.
Sclerocarya birrea (A. Rich.) Hochst.	Anacardiaceae	Udha	EL184	Kidney/ Birbirti	H	SB	F/D	CBdr
Secamone parvifolia (Oliv.) Bullock	Asclepiadaceae	Kiki	EL228	*Naqarssa*	L	L	F	CT
Senna occidentalis (L.) Link	Fabaceae	Hawacho	EL44	Loss of appetite /Albti kutu	H	SB	D	Cdr
				Gonorrhoea /Chobto	H	RB	F/D	CBdr.

Additional file 2 (continued)

Scientific name	Family	Local name	Herb. Vouch.	Disease treated (local name)	L/H	Part used	Form used	MPAP
Senna occidentalis (L.) Link	Fabaceae	Hawacho	EL44	Gonorrhoea /Chobto	H	R	F	CBdr.
Senna petersiana (Bolle) Lock	Fabaceae	Odusalem	EL172	Jaundice /Alati	H	L	F	CBdr
Senna septemtrionalis (Viv.) Irwin and Barneby	Fabaceae	Simamak	EL171	Hemorrhoids/ Qormade	H	RB	F/D	CRrb
				Swelling/Dula Binguge	H	L	F	CT
Sesamum indicum L.	Pedaliaceae	Selit	EL188	Mental disorder/Marata	H	Se	D	Cdr
Sida collina Schlechtend.	Malvaceae	Weed	EL127	Evil eye/ Buda	H	R	D	Cdr
Sida ovata Forssk.	Malvaceae	Urgosiwaka	EL126	Infections/Gemogi	L	RL	F/D	Cdr
Sida schimperiana A. Rich	Malvaceae	Misira	EL153	Impotence /Dhirumalebi	H	RL	D	CBdr.
Solanum giganteum Jacq.	Solanaceae		EL205	Diarrhoea /Bassa	H	R	F/D	CBdr.
Solanum incanum L.	Solanaceae	Hiddi	EL63	Headache/ Bowo	H	RB	F	Cex
				Impotence /Dhirumalebi	H	R	F	CBdr.
				Epilepsy in cattle/ Furtu	L	R	F	Cex
				Gonorrhoea/ Chobto	H	R	F	Cex
Steganotaenia araliacea Hochst. ex A. Rich.	Apiaceae	Boboftu	EL149	Jaundice /Alati	H	L	F/D	Cdr
Stephania abyssinica (Dillon and A. Rich) Walp.	Menispermaceae	Kalala	EL130	Vomit and fever/Dingetegna	H	R	F	Cdr.
				Jaundice /Alati	H	RL	F	Cdr
Stomatanthes africanus (Oliv. & Hiern) R. M. King and H. Rob	Asteraceae	Sara guracha	EL145	Headache/ Bowo	H	L	F/D	Cdr.
Suregada procera (Prain) Croizat	Euphorbiaceae	Xillo	EL185	Gonorrhoea/ Chobto	H	RB	D	CBdr
Syzygium guineense (Willd.) DC.	Myrtaceae	Unoma	EL39	Skinny cattles /Honoma	L	Rr	D	Cdr
				Urinary tract problem /Dukuba Finchani	H	RB	F/D	CBdr

Additional file 2 (continued)

Scientific name	Family	Local name	Herb. Vouch.	Disease treated (local name)	L/H	Part used	Form used	MPAP
Talinum caffrum (Thunb.) Eckl. and Zeyh.	Portulacaceae	Burka	EL224	Stabbing pain /Woransa	H	R	F/D	Cdr
Talinum portulacifolium (Forssk.) Asch. ex Schweinf.	Portulacaceae	Joshani	EL223	Hemorrhoids /Qormade	H	RB	D	CR
Tamarindus indica L.	Fabaceae	Roka	EL107	Tapeworm /Kosso	H	Se	F/D	CE
Terminallia brownii Fresen.	Combretaceae	Birdhesa	EL119	Jaundice/ Alati	H	RB	F	CB
Thalictrum rhynchocarpum Dill. & A. Rich.	Ranunculaceae	Bala/Sirebizu	EL535	Stabbing pain/Woransa	H	R	F/D	CBdr.
Tinnea somalensis Gurke ex Chiov.	Lamiaceae	Kertatumi	EL62	Ascariasis/ Magga	H	L	D	CBdr
Tinospora caffra (Miers) Troupin	Menispermaceae	Buri	EL74	Buda/ Evil eye	L	R	F	Cdr
Toddalia asiatica (L.) Lam.	Rutaceae	Kundo	EL97	Intestinal parasites/ Gara muru	H	L	F	CE
Tragia brevipes Pax	Euphorbiaceae	Lalesa	EL93	Urinary tract problem /Dukuba Finchani	H	R	F	CT
				Jaundice /Alati	H	R	F	Cdr
Trichilia dregeana Sond.	Meliaceae	Menisa	EL226	Gonorrhoea /Chobto	H	RB	D	CBdr
Trichilia emetica Vahl	Meliaceae	Botoro	EL90	Bassa/Diarrhoea	L	SB	D	Cdr
Trichodesma uniflora Brand	Boraginaceae		EL195	Sore /Sibiji	H	RB	F	CRrb
Triumfetta heterocarpa Sprague and Hutch.	Tiliaceae	Gurbi hola	EL146	Jaundice /Alati	H	R	F	CBdr
Vepris dainellii (Pchi-Serm) Kokwaro	Rutaceae	Arabe	EL110	Jaundice /Alati	H	R	F	CBdr
				Malaria/ Bussa	H	F	F	Cdr.
Vernonia amygdalina Del.	Asteraceae	Ebicha	EL22	Buda/ Evil eye	L	L	F	Cdr
Vernonia auriculifera Hiern.	Asteraceae	Ebicha	EL85	Diarrhoea /Tuma	L	L	F/D	Cdr

Additional file 2 (continued)

Scientific name	Family	Local name	Herb. Vouch.	Disease treated (local name)	L/H	Part used	Form used	MPAP
Vernonia myriantha Hook. f.	Asteraceae	Ragi	EL129	Abnormal menstruation /Dhiga	H	L	F	CT
Vernonia wollastonii S. Moore	Asteraceae	Hida gabro	EL86	Diarrhoea/Albati	H	RB	F	Cdr
				Mental disorder /Merata	H	RL	F/D	CBdr.
Vigna heterophylla A. Rich.	Fabaceae	Uhdusalem	EL124	Headache/ Bowo	H	R	F/D	CP
				Mental disorder /Merata	H	RL	F/D	CBdr.
Vigna membrenacea A. Rich.	Fabaceae	Ropi	EL163	Mental disorder /Ka marata	H	R	D	CE
Viscum tuberculatum A. Rich.	Viscaceae	Digelo	EL109	Jaundice/ Alati	L	L	F	Cdr.
Vitex doniana Sweet	Verbenaceae	Sari	EL220	Hepatitis/ Dukuba tiru	H	R	F/D	CR
				Kidney infection /Birbirti	H	R	F/D	CR
Warburgia ugandensis Sprague	Canellaceae	Befti	EL23	Headache /Bowo	H	SB	F/D	CP
				Pus from an ear /Dhukba guraa	H	SB	D	CR
Withania somnifera (L.) Dun.	Solanaceae	Unso	EL206	Swelling of nose in mule/ Chachabsa	L	R	F/D	CR
				Evil eye /Buda	H	R	D	CBdr.
				Epilepsy /Gagabdo	H	R	F/D	CBdr.
Woodfordia uniflora (A. Rich.) Koehne	Lythraceae	Itecha	EL181	Retained placenta /Hobati	L	L	F	Cdr
				Hepatitis/ Liver	H	R	F	CBdr.
Xanthium strumarium L.	Asteraceae	Konti katiro	EL5	Tenia versicolor /Barley	H	L	F	Crb
Ximenia americana L.	Olacaceae	Uhdusalem	EL50	Evil eye /Buda	L	R	F	Cdr/Cp
				Impotence/ Dhirumalebi	H	R	F	CT
Ximenia caffra Sond.	Olacaceae	Hudha	EL174	Evil spirit /Jinni	H	R	F/D	CR
Zanthoxylum usambarense (Engl.) Kokowara	Rutaceae	Geda	EL98	Kidney infection/ Birbirti	H	SB	D	CBdr.
				Swelling of joints/ Gofla	L	SB	F	CT

Additional file 2 (continued)

Scientific name	Family	Local name	Herb. Vouch.	Disease treated (local name)	L/H	Part used	Form used	MPAP
Zinnia peruviana L.	Asteraceae		EL240	Diarrhoea /Bassa	H	R	F/D	CBdr.
Ziziphus abyssinica Hochst. ex A. Rich.	Rhamnaceae	Kankura	EL8	Jaundice/ Alati	H	RL	F	Cdr
*Zornia apiculat*a Milne-Redh.	Fabaceae	Kumbishi	EL234	Dhirumalebi/Impotence	H	RB	D	CBdr

Keys
Part used: Leaf, L.; Root, R; Fruit, F; Bark, B; Root bark, RB; Stem Bark, SB. For Livestock or human (L/H).
Form used: Dry, D; Fresh, F).
Methods of preparation and application (MPAP)_ Crush and tie, CT; Crush boil and drink; CBdr; Cdr, Crush and drink; Crush and extract its content, Cex; Crush and paint, Cpa.
